# Venous Thrombosis Associated with Different Types of SARS-CoV-2 Vaccines in the Netherlands—Results of the TERA Case-Control Study

**DOI:** 10.1055/a-2665-2400

**Published:** 2025-08-08

**Authors:** Willian J. van Dijk, Agnes C. Kant, Astrid van Hylckama Vlieg, Frits R. Rosendaal

**Affiliations:** 1 Department of Clinical Epidemiology, Leiden University Medical Center, Leiden, The Netherlands; 2 The Netherlands Pharmacovigilance Centre Lareb, 's-Hertogenbosch, The Netherlands

**Keywords:** venous thrombosis, SARS-CoV-2 vaccines, case control, population attributable fraction

## Abstract

**Background:**

The magnitude of the risk of venous thromboembolism (VTE) after SARS-CoV-2 vaccines is debated.

**Methods:**

We included patients with a first VTE in 2021 and controls from a sample of Dutch citizens. Participants completed a questionnaire on VTE risk factors and vaccination, with data linked to Statistics Netherlands. Odds ratios (OR) with 95% confidence intervals (95%CI) expressed the relative rate of VTE within 28 days post-vaccination, adjusted for age, sex, BMI, month of index date, and major VTE risk factors (COVID-19, surgery, cancer, and immobilization). Using previously reported age-stratified VTE incidences, we estimated vaccination's net impact by comparing the number of events attributed to vaccination and prevented by vaccine-induced protection against COVID-19-associated VTE.

**Results:**

We included 779 VTE patients and 5,311 controls. mRNA vaccines were not associated with VTE risk (BNT162b2 [Pfizer- BioNTech] vaccine OR 1.0, 95%CI 0.7–1.3; mRNA-1273 [Moderna] vaccine OR 1.4, 95%CI 0.8–2.4). Vector-based vaccines were associated with VTE risk (AZD1222 [AstraZeneca]: OR 1.5, 95%CI 1.0–2.5; Ad26.COV2.S [Johnson & Johnson]: OR 2.9, 95%CI 0.9–9.2). Excluding participants with major VTE risk factors, risks changed (BNT162b2: OR 1.5, 95%CI 1.1–2.1; mRNA-1273: OR 0.8, 95%CI 0.3–2.3; AZD1222: OR 2.0; 95%CI 1.0–3.9; and Ad26.COV2.S: OR 3.4; 95%CI 0.7–15.5). We estimated that SARS-CoV-2 vaccines contributed to approximately 700 VTEs but prevented approximately 3,700 VTEs.

**Conclusion:**

SARS-CoV-2 vaccines are associated with VTE, with varying risks between types of vaccines, and by sex and age. On a population level, in the Netherlands in 2021, SARS-CoV-2 vaccination resulted in a net benefit for the number of VTE events.

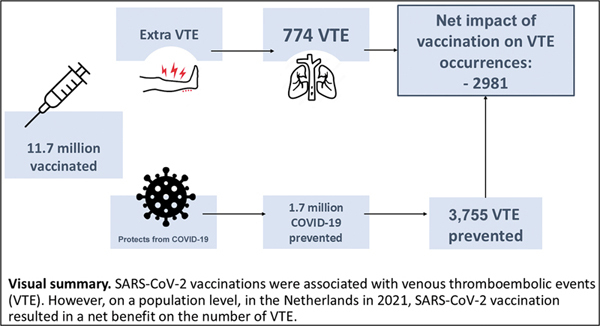

## Introduction

During the year 2021, over 9 billion SARS-CoV-2 vaccines were administered worldwide, which were estimated to have prevented over 10 million deaths. 1 2 However, soon after the start of the vaccination campaign, several cases of venous thromboembolisms (VTEs) following SARS-CoV-2 vaccination were reported. 3 4 5 This included reports of common manifestations of VTE, i.e., deep vein thrombosis and pulmonary embolism, which have a background incidence of 1 to 2 per 1,000 per year, as well as an extremely rare (<1 per 25,000 vaccine doses) form of VTE characterized by thrombocytopenia, which has been called vaccine-induced immune thrombotic thrombocytopenia (VITT). 6 7 These severe side effects were not detected in the primary randomized controlled trials (RCTs), because these RCTs were underpowered for this rare event. 8

VTE is a relatively common and impactful disorder, and especially the reports on VITT led to changes in the vaccination strategy in many countries. 9 For example, in the Netherlands, vaccination with the AZD1222 (AstraZeneca) vaccine was first paused and later discontinued in individuals younger than 60 years after reports of VITT in Denmark and Norway. 10 In addition a higher than expected number of spontaneous reports of VTE was received relative to background incidences. 11 Subsequently, large register-based studies showed increased rates of VTE following SARS-CoV-2 vaccines, in rare cases with thrombocytopenia but far more often without. 3 12 13 14 15 16 17 18 19 20 21 22 This association was present particularly in, but not limited to, the vector-based vaccines (AZD1222 [AstraZeneca] and Ad26.COV2.S [Johnson & Johnson]). 23 In contrast, other studies showed no association between SARS-CoV-2 vaccines and VTE. 13 20 21 24 25

Many previous reports on the association between SARS-CoV-2 vaccines and VTE used self-controlled case series (SCCS) design. 13 14 21 26 27 28 One of the assumptions of this design is that the outcome (VTE) should not influence the subsequent probability of the exposure (vaccination). 26 In this specific situation, this assumption is unlikely to hold, i.e., a VTE may prompt the likelihood of subsequent vaccination. Although there are methodological solutions for this in the SCCS design, other study designs may be better suited to provide a valid estimate of the VTE risk associated with SARS-CoV-2 vaccines.

To study the risk of VTE associated with different SARS-CoV-2 vaccines, we performed a case-control study including patients with a first VTE in 2021 and controls without VTE, i.e., The Thrombosis Etiology and Risk factor Assessment (TERA) study. In addition to the overall risk estimation, we assessed the risk of VTE after SARS-CoV-2 vaccination in different subgroups, defined by the presence or absence of additional VTE risk factors. Finally, we estimated the absolute number of VTE events attributed to and prevented by SARS-CoV-2 vaccinations in the Netherlands in 2021, to provide an estimate of the net effect of the vaccination campaign on VTE risk.

## Methods

### Study Design

Patients were selected from the files of 10 participating hospitals in the Netherlands (Leiden, The Hague, Amsterdam, Rotterdam (two), Nijmegen (two), Nieuwegein, Groningen, Eindhoven). We included patients who had a first VTE in 2021 based on diagnostic codes (DBC code: diagnosis–treatment combination) for pulmonary embolism or (deep) venous thrombosis. The selection of patients was performed between October 2022 and August 2023 using the diagnostic codes as listed in Supplementary Material A2 (available in the online version). In eight of the hospitals an opt-in procedure was used, in which patients were invited to participate. After informed consent was obtained, patients were sent a detailed questionnaire containing questions on the VTE, comorbidities, VTE-related risk factors, and vaccination status (date, dose, and type of vaccine) ( Supplementary Material B , available in the online version). In two hospitals (Leiden and The Hague), participants were invited via an opt-out procedure. Patients recruited from these hospitals received the questionnaire directly, without prior consent.

Controls were recruited from a population-based longitudinal cohort of 7,000 randomly selected Dutch citizens from the online LISS (Longitudinal Internet studies for the Social Sciences) panel administered by Centerdata (Tilburg University, the Netherlands). 29 Individuals were not eligible as controls if they had suffered a VTE. In addition, we excluded controls with low-quality data: when they had an unrealistic completion time (under 3 minutes; <5th percentile completion time) in combination with exclusively negative answers.

The questionnaire was sent to both the patients and the controls and was filled in between 2022 and 2023. The questionnaire was provided in Dutch or English, depending on the background of the participant, and could be filled in on paper or digitally depending on the preference of the participant. Importantly, the questionnaire was presented as a study on general risk factors for VTE and not specific for SARS-CoV-2 vaccination. With information provided by the participants in the questionnaire, the first VTE defined by the hospital diagnostic code, or the absence thereof, was verified. Participants filling in at least 30% of the questions (including the primary exposure: SARS-CoV-2 vaccination) were included in the analyses.

Data of participants were enriched with healthcare information by linking them to data from Statistics Netherlands (CBS) for participants who consented to this linking. Informed consent for use and linking of their data was obtained digitally or in writing from all participants, at the start of the questionnaire.

### Definitions

For the main analyses, we included patients who were alive at the time of the selection procedure, and were able to fill in the questionnaire. Confirmation of a first VTE was based on data from the questionnaire or CBS and when the event was not a first event but rather a recurrence, this patient was excluded from the analysis.

We defined the exposure, i.e., type of SARS-CoV-2 vaccination, as the vaccine registered in the data from CBS. The National Institute for Public Health and the Environment collects COVID-19 vaccination data on a national level in the COVID Vaccination Information and Monitoring System (CIMS) for all persons who gave consent to share their data in CIMS (≈94%). 30 31 The CIMS database contains vaccinations that were administered by diverse institutions, for example, Municipal Health Services, general practitioners, and nursing homes, but is not entirely complete. Therefore, if no vaccination was registered in CIMS, we additionally used data from the questionnaire. In a similar way, we gathered information on having had a SARS-CoV-2 infection, i.e., data from CBS, and, in absence of information in CBS, supplemented it with data from questionnaires.

To assess the risk of VTE associated with SARS-CoV-2 vaccination, we defined the risk period of a vaccination-associated VTE to be up to 28 days after vaccination. We therefore assessed whether patients and controls were vaccinated in the 28 days prior to the index date. For patients, the index date was the date of VTE diagnosis; for controls, a random index date in the year 2021 was generated. In the analysis on the risk of VTE associated with a specific vaccine dose, i.e., first or second, the most recent vaccine dose relative to the index date was used.

In addition, we assessed the risk of VTE associated with SARS-CoV-2 vaccination in several subgroups, defined by the presence or absence of additional VTE risk factors. We focused on the major VTE risk factors, i.e., cancer (diagnosed less than 5 years ago), surgery in the past 90 days, COVID-19 in the past 60 days, and immobilization for at least 3 days in the past 90 days (see Supplementary Material A4 (available in the online version) and Kearon et al 32 ). We considered a VTE risk factor to be present when it was mentioned in the questionnaire or when it was registered in the healthcare data within CBS (from three datasets: two type of diagnostic codes and medication prescriptions; see Supplementary Materials A1 and A5 , available in the online version). To prevent counting diagnoses of “suspicion of cancer” as a cancer diagnosis, we considered a diagnosis for cancer present when it was registered in multiple data sources within CBS or when it was mentioned in the questionnaire.

### Statistical Analysis

Demographics of patients and controls were given as proportions or means. Self-reported weight and height were used to calculate body mass index (BMI). To estimate the relative risk of VTE, we used odds ratios, after adjustment for potential confounding factors. In addition to age, BMI (both as a continuous variable), and sex (categorical), we adjusted for calendar time with month of index date as a covariate in the multivariable logistic regression analyses. As endpoints we analyzed all VTEs as well as deep vein thrombosis (DVT) and pulmonary embolism (PE) separately.

Further analyses assessed risks for different SARS-CoV-2 vaccines (type and dose), and analyses in subgroups by sex and age (≤60 year; >60 year). We performed a sensitivity analysis in which different risk periods were applied, i.e., in addition to defining a vaccination-associated VTE as a VTE occurring within 28 days after vaccination, we also used time periods of 14 and 180 days.

In the analysis on the combined effect of VTE risk factors and vaccination, only participants with non-missing data on BMI (0.1% missing) and VTE risk factor (3% missing) were analyzed (complete case analysis). We performed two sensitivity analyses for each VTE risk factor with missing information, assuming extreme answers when information on risk factors was missing (all missing values imputed as “risk factor present” or “risk factor not present”). In addition, we performed two sensitivity analyses regarding the risk factor assessment: rather than combining both data sources, we analyzed the data once using only risk factor data from CBS and once using only risk factor data from the questionnaire.

To estimate the impact of vaccination on VTE occurrence in 2021 in the Netherlands, we estimated the absolute number of events attributed to vaccination as well as the absolute number of events prevented by vaccine-induced protection against COVID-19 associated VTE. We stratified these calculations for age, i.e., ≤60 years of age and >60 years of age.

To estimate the absolute number of VTE events attributed to SARS-CoV-2 vaccines, we first calculated the population attributable fraction (PAF) for each type of vaccine. The PAF is the fraction of total VTE events attributed to this specific exposure. For the calculation of the PAF, we used the formula: PAF = pd × ([aOR - 1]/aOR), in which pd is the proportion of cases exposed to a type of vaccine and aOR is the OR (adjusted for time, sex, BMI, age, and the four VTE risk factors) for that type of vaccine. 33 34 To calculate the absolute number of VTE events attributed to these vaccines, the PAF was multiplied by the number of registered VTE evens in healthcare data of CBS in 2021.

Subsequently, we estimated the number of VTE events attributed to SARS-CoV-2 infection stratified by vaccination status. We considered an individual vaccinated if a vaccine was administered between 6 and 2 months prior to SARS-CoV-2 infection (2 months to prevent inclusion of vaccines causing VTE and 6 months because of waning effectiveness of vaccines for symptomatic SARS-CoV-2 infection). 35 We used the same formula for the PAF (in which pd now is the proportion of cases with a SARS-CoV-2 infection with or without prior vaccination, and the aOR is the relative risk of VTE after (un)vaccinated SARS-CoV-2 infection). This PAF was multiplied by number of registered VTE events in CBS in 2021, resulting in the number of VTE events following (un)vaccinated SARS-CoV-2 infections. Subsequently, we calculated the percentage of individuals with (un)vaccinated SARS-CoV-2 infections who developed a VTE event.

To estimate the number of vaccine-prevented SARS-CoV-2 infections we used the formula: PC = vaccinated SARS-CoV-2 infections in CBS × (1/[1 − VE]), in which PC is the number of SARS-CoV-2 infections that was prevented and VE is the vaccine effectiveness for SARS-CoV-2 infection, assuming situations with VE 50, 75, and 90%. 36 37 The number of prevented SARS-CoV-2 infections was multiplied by the ratio of VTE events following unvaccinated SARS-CoV-2 infections, resulting in the hypothetical number of SARS-CoV-2–related VTE that would have occurred without vaccination in 2021. Using the number of VTE attributed to the vaccines and the hypothetical number of prevented VTE after SARS-CoV-2 without vaccination in 2021, the net number of VTE attributed to SARS-CoV-2 vaccines was estimated.

In an a priori power analysis with a power of 80%, an alpha of 5% and an exposure rate in the control group of 10% with 5 controls per case, at least 160 patients were needed to be included to detect an odds ratio of 2. The sample size for the analyses per type of vaccine (with a minimal exposure rate of 2%) was 650 patients for an odds ratio of 2. Statistical analyses were carried out with STATA 16.1 for Windows (StataCorp, College Station, USA).

## Results

**Fig. 1:**
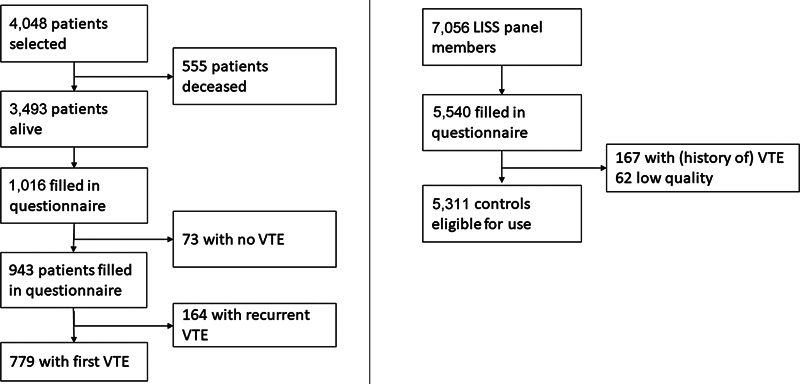
Flowchart of inclusion of eligible patients and controls. LISS, Longitudinal Internet studies for the Social Sciences; VTE, venous thromboembolism.

In total, 4,048 VTE patients were selected from the files of the 10 Dutch hospitals. Of these patients, 555 (14%) were deceased before selection. Of the 3,493 living patients who were invited to participate, 1,016 (29%) returned the questionnaire. Of these 1,016 patients, 744 (73%) gave permission for linkage to data of CBS, which was successful in 716 (96%). Of the 1,016 individuals who returned a questionnaire, 779 were eligible as cases (see Fig. 1 ). Of the patients who were eligible, 579 patients gave permission for linkage and were successful linked to data of CBS (74%). In the control group, from the population-based longitudinal cohort of randomly selected Dutch citizens, 5,540 out of 7,056 (79%) individuals returned the questionnaire. From the total 5,311 eligible controls (see Fig. 1 ), 4,709 (89%) participants were successfully linked to data of CBS.

Demographics of the patients and controls are summarized in Table 1 . Patients were older, more frequently male, and had a higher BMI than the control subjects. Most of the VTE events were pulmonary emboli (54%), 35% were DVT, and 11% were from atypical origin (such as VTE in the arm, portal vein, or cerebral venous sinus). More information about the timing and distribution of the SARS-CoV-2 vaccinations in the Netherlands is summarized in the Supplementary Materials A7 and A8 (available in the online version). Most of the vaccines were administered during the Spring of 2021 and the most commonly administered vaccine was BNT162b2 (Pfizer- BioNTech). The AZD1222 (AstraZeneca) vaccine was predominantly administered to individuals aged between 60 and 70 years of age and the Ad26.COV2.S (Johnson & Johnson) vaccine to individuals aged below 60 years of age.

**Table 1:** Demographics of included controls and patients with a first venous thromboembolic event

		Controls	Patients
**Total**		5,311	779
**Age**	Mean (SD)	53.5 (18.2)	61.1 (14.1)
**Sex**	Men, *n* (%)	2,476 (46.6)	438 (56)
**BMI**	Mean (SD)	26.0 (4.7)	27.7 (5.2)
	Missing, *n* (%)		2 (0.3)
**VTE type**	Pulmonary embolism, *n* (%)	–	422 (54)
	DVT leg, *n* (%)	–	273 (35)
	Other, *n* (%)	–	84 (11)
**Risk factor**	Cancer (<5 years), *n* (%)	195 (3.7)	102 (14)
	Immobilization (<90 days), *n* (%)	99 (1.9)	209 (28)
	Surgery (<90 days), *n* (%)	125 (2.4)	110 (15)
	COVID-19 (<60 days), *n* (%)	167 (3.1)	113 (15)
	Any (of the above), *n* (%)	513 (9.7%)	352 (45%)
	None, *n* (%)	4,674 (88)	311 (40)
	Missing, *n* (%)	124 (2.3)	116 (15)

Abbreviations: BMI, body mass index; COVID-19, corona virus disease 2019; DVT, deep venous thrombosis; SD, standard deviation.

In the main analysis, when a SARS-CoV-2 vaccine exposure occurred within 28 days prior to VTE, the mRNA-type vaccines were not, or at the most mildly, associated with an increased risk of VTE (BNT162b2 [Pfizer]: aOR 1.0 [95%CI 0.7, 1.3] and mRNA-1273 [Moderna]: aOR 1.4 [95%CI 0.8, 2.4]; see Table 2 ). The vector-based vaccine types were associated with an increased VTE risk (AZD1222: aOR 1.5 [95%CI 1.0, 2.5] and Ad26.COV2.S aOR 2.9 [95%CI 0.9, 9.2]; see Table 2 ). In the sensitivity analysis, when a vaccination-associated VTE was defined as a VTE occurring within 14 or within 180 days after vaccination, relative risks were highest in the first 14 days after vaccination (14 days: AZD1222: aOR 1.8 [95%CI 1.0, 3.3] and Ad26.COV2.S aOR 3.8 [95%CI 1.0, 15.4]; see Supplementary Table S1A–C , available in the online version).

**Table 2:** Odds ratios for the association between the type and dose of vaccination and the risk of a first venous thromboembolic event within 28 days

				mRNA						Vector					
28-day risk period		Any first VTE		BNT162b2 (Pfizer- BioNTech)			mRNA-1273 (Moderna)			AZD1222 (AstraZeneca)			Ad26.COV2.S (Johnson & Johnson) a		
Any vaccine dose		Cases	Controls	aOR b	95% CI		aOR	95% CI		aOR	95% CI		aOR	95% CI	
	**All**	779	5,311	1.0	0.7	1.3	1.4	0.8	2.4	1.5	1.0	2.5	2.9	0.9	9.2
	**Men**	438	2,476	0.8	0.6	1.2	1.7	0.9	3.3	1.3	0.7	2.6	4.4	1.1	18.1
	**Women**	341	2,835	1.2	0.8	1.7	1.1	0.5	2.5	1.8	0.9	3.7	1.6	0.2	13.7
	**≤60**	327	3,118	1.1	0.7	1.6	1.0	0.4	2.6	2.0	0.7	5.7	3.5	1.1	11.4
	**>60**	452	2,193	0.9	0.6	1.4	1.5	0.8	2.9	1.2	0.7	2.1			
First vaccine dose	**All**	779	5,311	1.1	0.8	1.6	2.4	1.1	5.5	1.8	1.0	3.3	3.0	0.9	9.3
	**Men**	438	2,476	1.0	0.6	1.7	3.4	1.2	9.4	1.4	0.6	3.3	4.5	1.1	18.7
	**Women**	341	2,835	1.2	0.7	2.0	1.3	0.3	5.8	2.3	1.0	5.4			
	**≤60**	327	3,118	1.2	0.7	2.2	0.9	0.2	4.0	3.0	0.9	10.4	3.5	1.1	11.6
	**>60**	452	2,193	1.1	0.7	1.8	5.4	1.7	17.3	1.3	0.6	2.6			
Second vaccine dose	**All**	779	5,311	0.9	0.6	1.3	1.5	0.6	4.1	1.2	0.5	2.0			
	**Men**	438	2,476	0.7	0.4	1.2	2.7	0.8	8.8	1.1	0.4	2.2			
	**Women**	341	2,835	1.1	0.6	1.8	0.6	0.1	4.6	1.2	0.4	2.5			
	**≤60**	327	3,118	0.9	0.5	1.6	1.2	0.3	4.1						
	**>60**	452	2,193	0.9	0.5	1.5	1.8	0.3	9.0	1.0	0.4	1.9			

Abbreviation: VTE, venous thromboembolic event.

a The Johnson & Johnson vaccine was administered only once per person.

b aOR, adjusted odds ratio; adjusted for age, sex, Body Mass Index (BMI), calendar time (month).

Relative risks varied between age groups and sexes. The AZD1222 vaccine was associated with VTE predominantly in young (<60 years: aOR 2.0 [95%CI 0.7, 5.7]) and in women (aOR 1.8 [95%CI 0.9, 3.7]). The Ad26.COV2.S vaccine, which was administered only to individuals aged <60 years old, was associated with a high relative risk of VTE in men (aOR 4.4 [95%CI 1.1, 18.1]).

The risk of VTE differed per vaccine dose. For the vaccines associated with VTE risk (except for the Ad26.COV2.S vaccine which is administered only once), the risk of VTE was higher for the first dose than for the second dose (mRNA-1273 first dose aOR 2.4 [95%CI 1.1, 5.5], second dose aOR 1.5 [95%CI 0.6, 4.1]; AZD1222 first dose aOR 1.8 [95%CI 1.0, 3.3], second dose aOR 1.2 [95%CI 0.5, 2.0]).

SARS-CoV-2 vaccination affected the occurrence of both DVT and PE (see Supplementary Table S1A–C , available in the online version). The Ad26.COV2.S vaccine showed an association with the risk of both PE and DVT (aOR PE: 3.8, 95%CI 1.1, 14 and aOR DVT: 2.1, 95%CI 0.3, 16.3). The AZD1222 vaccine was predominantly associated with DVT (aOR DVT 1.8, 95%CI 0.9, 3.7 and PE 1.3, 95%CI 0.7, 2.5).

We subsequently stratified according to the presence or absence of other VTE risk factors as shown in Table 3 . The presence of any of the included risk factors for VTE, i.e., cancer (diagnosed less than 5 years ago), surgery in the past 90 days, and immobilization for at least 3 days in the past 90 days, or COVID-19 in the past 60 days, was associated with a 10.8-fold increased risk of VTE (95%CI: 8.8, 13.2). In the absence of VTE risk factors, the AZD1222 and Ad26.COV2.S vaccines were still associated with VTE risk (AZD1222: aOR 2.0, 95%CI 1.0, 3.9 and Ad25.COV2.S: aOR 3.4, 95%CI 0.7, 15.5), while the risk appeared mildly increased for the BNT162b2 vaccine (BNT162b2: 1.5, 95%CI 1.1, 2.1) and not for the mRNA-1273 vaccine (aOR 0.8, 95%CI 0.3, 2.3). The combination of VTE risk factors and SARS-Cov-2 vaccination was not associated with a markedly further increased risk of VTE for any vaccine except Ad26.COV2.S (aOR 35.0, 95%CI 3.0, 1414.6), albeit confidence intervals were wide. See Supplementary Table S2 (available in the online version) for combined effect of vaccines and individual VTE risk factors. Sensitivity analyses assuming extreme values for missing data about VTE risk factors or with only risk factor data from CBS or the questionnaire resulted in some changes in the estimates, but did not alter conclusions (see Supplementary Table S2A–D , available in the online version).

**Table 3:** Odds ratios describing association among venous thromboembolic event, venous thromboembolic event risk factors, and vaccines in the past 28 days

Risk factor a	Vaccination	BNT162b2 (Pfizer- BioNTech)	mRNA-1273 (Moderna)	AZD1222 (AstraZeneca)	Ad26.COV2.S (Johnson & Johnson)
–	–	1 [ref]	1 [ref]	1 [ref]	1 [ref]
+	–	10.8 (8.8–13.2)	10.8 (8.8–13.2)	10.8 (8.8–13.2)	10.8 (8.8–13.2)
–	+	1.5 (1.1–2.1)	0.8 (0.3–2.3)	2.0 (1.0–3.9)	3.4 (0.7–15.5)
+	+	6.2 (3.7–10.5)	15.1 (6.5–34.9)	12.1 (4.8–30.2)	35.0 (3.0–1414.6)

a Cancer diagnosis not longer than 5 years ago, immobilization for at least 3 days in the past 90 days, surgery in the past 90 days, COVID-19 infection in the past 60 days adjusted for age, sex, and month; 659 cases (349 [53%] with venous thromboembolic event risk factor) and 5,177 controls (511 [10%] with venous thromboembolic event risk factor).

In addition to the relative risk of VTE after SARS-CoV-2 infections, we made an estimate of the absolute effect of vaccination in the context of a pandemic in the Netherlands in 2021. During that year, in the Netherlands approximately 22,500 VTE events were registered (with an overall annual incidence of 0.16%), of which 7,500 (annual incidence: 0.08%) occurred in individuals aged 60 or younger and approximately 15,000 (annual incidence: 0.32%) in individuals older than 60 years old. A total of 11 million individuals received one of the SARS-CoV-2 vaccines. The PAF of VTE of all SARS-CoV-2 vaccinations combined was 5.1% for individuals younger and 2.6% for individuals older than 60 years old. Based on this PAF and the absolute number of VTE registered, we estimated that 385 (95%CI −562, 781) VTE events in individuals aged <60 years and 389 (95%CI −1,429, 1,402) VTE events in >60 years were attributed to SARS-CoV-2 vaccines (see Table 4 and details in Supplementary Table S3 , available in the online version). However, vaccination also prevented SARS-CoV-2 infections, and subsequent VTE events. Assuming a vaccine effectiveness of 75%, vaccination prevented over 1.3 million SARS-CoV-2 infections in individuals aged <60 and over 300,000 infections in persons aged >60. This would have resulted in 1,274 (95%CI 1,164; 1,342) VTE evens in individuals younger than 60 years and 2,481 (95%CI 2,306; 2,581) VTE events in individuals older than 60 years. These numbers on VTE events attributed to vaccinations and VTE events prevented by vaccinations lead to, on a population level, prevention by vaccination of over 800 VTE events in individuals <60 years and over 2,000 in individuals older than 60 years, with a net beneficial effect of SARS-CoV-2 vaccines on VTE incidence (see Table 4 ). Assuming a lower vaccine effectiveness, the net benefit of vaccination persists, even with a vaccine effectiveness as low as 50%.

**Table 4:** Absolute number (95% confidence intervals) of venous thromboembolic events caused and prevented by SARS-CoV-2 vaccines

	Population (adult)	VTE in 2021	
≤60 years	9,568,997	7,562	
>60 years	4,597,642	14,854	
**Population attributable fraction**	Vaccination	COVID-19 vaccinated	COVID-19 unvaccinated
≤60 years	5.1% (−7.4; 10.3)	0.3% (−2.3; 1.0)	13.0% (11.9; 13.7)
>60 years	2.6% (−9.6; 9.4)	1.9% (1.1; 2.3)	11.1% (10.3; 11.5)
**Number of:**	Vaccination	COVID-19 vaccinated	COVID-19 unvaccinated
≤60 years	7,531,592 (79%)	341,622 (3.6%)	1,054,177 (11.0%)
>60 years	4,185,689 (91%)	92,139 (2.0%)	244,510 (5.3%)
**VTE associated with:**	Vaccination	COVID-19 vaccinated	COVID-19 unvaccinated
≤60 years	385 (−562; 781)	20 (−171; 74)	983 (898; 1,035)
>60 years	389 (−1,429; 1,402)	288 (162; 34)	1,646 (1,530; 1,713)
**Proportion with VTE**	Vaccination	COVID-19 vaccinated	COVID-19 unvaccinated
≤60 years	0.005%	0.006%	0.093%
>60 years	0.009%	0.312%	0.673%
**SARS-CoV-2 infections prevented**			
Assumed vaccine effectiveness	50%	75%	90%
≤60 years	683,244	1,366,488	3,416,220
>60 years	184,278	368,556	921,390
**VTE prevented by vaccines**			
≤60 years	637 (582; 671)	1,274 (1,164; 1,342)	3,184 (2,910; 3,354)
>60 years	1,240 (1,153; 1,291)	2,481 (2,306; 2,581)	6,202 (5,766; 6,454)
**Net effect of vaccination on VTE**			
≤60 years	−251 (−1,233; 199)	−888 (−1,903; −383)	−2,799 (−3,916; −2,130)
>60 years	−851 (−2,719, 248)	−2,092 (−4,010; −905)	−5,813 (−7,882; −4,364)

Abbreviation: VTE, venous thromboembolic event.

Note: See Supplementary Table S3 (available in the online version) for more details.

## Discussion

The aim of this study was to assess the risk of VTE associated with SARS-CoV-2 vaccines. Our results indicate that mRNA-type vaccines were not, or at the most mildly, associated with an increased risk of VTE. The vector-based vaccines were associated with an increased risk of VTE, with relative risk estimates ranging from 1.5 for the AZD1222 vaccine to 2.9 for the Ad26.COV2.S vaccine. The risk of VTE varied by sex and age, depending on the type of vaccine.

The increased relative risk of VTE indicates that vaccines potentially lead to an increase in the absolute number of VTE events in the population. However, the protection against SARS-CoV-2 infection, which in itself is a risk factor for VTE, will prevent VTE events. Therefore, we assessed the net effect of SARS-CoV-2 vaccinations on VTE occurrence. Regardless of age, SARS-CoV-2 vaccines had a net beneficial effect on the number of VTE events, i.e., vaccines prevented more VTE events than they caused. Of course, this calculation concerns the effect on VTE only, while vaccination also has other major advantages. 35

In the absence of VTE risk factors, the mRNA-1273 vaccine was not associated with the risk of VTE. The increased risk after this vaccine in the overall analyses may be explained by the preferential vaccination of individuals with risk factors with this vaccine. Indeed, in the Netherlands, this vaccine was predominantly used in older and frail persons, i.e., those with an increased VTE risk. 31 38 In contrast, the BNT162b2 vaccine was associated with a mildly increased risk of VTE in the absence of VTE-related risk factors but not in the overall study population. In both circumstances (residual) confounding may have played a role.

To our knowledge this is the first case-control study estimating the relative risks for VTE after all types of SARS-CoV-2 vaccination using detailed information from multiple sources and adjustment for multiple confounding factors. In line with our result, many prior studies found an increased risk of VTE after SARS-CoV-2 vaccines. 3 14 15 19 22 27 39 40 The relative risk was highest after vector-based vaccines, 14 17 19 22 27 39 40 although some studies have also reported an increased risk for the BNT162b2 vaccine. 14 16 40 Comparing relative risks for VTE after SARS-CoV-2 vaccines, unadjusted for possible confounding VTE risk factors, is difficult, because of differences between countries in vaccination strategies. Countries differed in timing and type of vaccines administered to specific targeted populations, resulting in different impact of confounding per country. 41 The net protective effect of vaccination on (COVID-19 related) VTE was also confirmed in prior research. 42 43

The major strength of our study is the detailed data available about VTE risk factors (both self-reported and from different registries) and vaccination. Using these data, we were able adjust for many possible confounders. We combined self-reported data and registry-based data, which limited the impact of both recall bias and registry bias. We included cases from several hospitals across the country and the control group consisted of a random sample of Dutch citizens.

Our study also has limitations. Most of VTE cases were selected using hospital diagnostic codes, which may have resulted in missing less severe VTE cases or cases with a second, more important, diagnosis. However, in the Netherlands, all PE and most DVT are treated (or diagnosed) in hospitals. The response rate of the patients was low (29%), which may have resulted in inclusion of patients who were healthier than average. The higher response rate in the control group was most likely due to participants from the LISS panel being more accustomed to regularly completing questionnaires. It is possible that individuals who are part of such a panel exhibit above-average health-seeking behaviors, and are therefore more likely to receive vaccinations. In addition, the linkage with data from CBS was less successful for cases compared to controls. Both these effects may have led to an underestimation of the true effect of SARS-CoV-2 vaccination on VTE in our study. Unfortunately, the study was underpowered for detailed subgroup analyses (for example, individuals with a minor VTE risk factor, such as hormonal contraceptives or atypical VTE, such as cerebral venous sinus thrombosis). Furthermore, sample size was small for the Ad26.COV2.S vaccine (the least frequently administered vaccine in the Netherlands). There were no blood measurements performed in this study; therefore, we could not study the effects of vaccination on coagulation parameters. However, several prior studies found small and transient increase in coagulation parameters. 44 45 46 We were unable to test for specific booster effects, because boosters were administered predominantly after the inclusion period of our study. In estimating the absolute number of VTE cases caused or prevented by SARS-CoV-2 vaccination, we could have compared the number of VTE cases as registered in CBS in 2019 to those in 2021. However, this approach would not allow us to differentiate between VTE cases attributable to SARS-CoV-2 vaccines and those resulting from SARS-CoV-2 infections, nor detect preventing excess cases. Moreover, numerous other factors changed between 2019 and 2021, such as public health measures, a reduced incidence of other infections, and more. Instead, by using the PAF, we were able to account for these confounding factors and provide a more accurate estimation. Furthermore, for this calculation, we assumed that the effectiveness of vaccination was consistent in both magnitude and duration, irrespective of the type of vaccine used. In addition, these calculations are dependent on several contextual factors, e.g., status of the pandemic.

In conclusion, SARS-CoV-2 vaccines are associated with the risk of VTE after extensive adjustment for confounders, which varied between types of vaccines, sexes, and age. Highest risks were seen in vector-based vaccine types. On a population level, in the Netherlands in 2021, SARS-CoV-2 vaccines had a net beneficial effect on the number of VTE events.

## Notes

**What is known about this topic?**

- Varying risks of VTE after SARS-CoV-2 vaccines are reported.

**What does this paper add?**

- SARS-CoV-2 vaccines are associated with VTE, though the association is confounded by underlying risk factors (for both VTE and severe COVID-19).
- On a population level, in the Netherlands in 2021, SARS-CoV-2 vaccination resulted in a net benefit for the number of VTE events.
