# Supplementary material for: Venous Thrombosis Associated with Different Types of SARS-CoV-2 Vaccines in the Netherlands—Results of the TERA Case-Control Study
Source: Thromb Haemost. 2025 Aug 8;126(6):636–45. doi: 10.1055/a-2665-2400 (PMC13288348; doi:10.1055/a-2665-2400)
Supplement: Supplementary file 1 — Supplementary Material [file 10-1055-a-2665-2400-s25030132.pdf]

Supplement Document A

Content

1-Used Data Sources 2
2-Selection of True Cases 2
3-Selection of True Controls 2
4-Definition VTE Risk factor 3
5-Used Codes in Dutch Statistics 4
6-Method of dealing with (partly) missing values in questionnaire data 5
7-Description of population having a VTE in 2021 5
8-Description of population vaccinated in 2021 in the Netherlands 7

1-Used Data Sources

1. Local hospital billing data – selection of cases
2. Local hospital VTE specific data – selection of cases
3. Questionnaire
4. Dutch Statistics
   a. SARS-CoV-2 vaccination dataset (CIMS)
   b. SARS-CoV-2 test result dataset (GGDCOVID19BM)
   c. Hospitalization dataset; with diagnoses (DHD; LBZBASIS/LBZDIAGNOSEN; ICD10)
   d. Billings dataset (VEKTIS MSZZORGPRESTATIES/Subtrajecten; DBC)
   e. Medication reimbursed dataset (Medicijntab, atc4)
   f. Causes of death dataset (DOODOORZ)

2-Selection of True Cases

All cases were selected in 10 Dutch hospitals using billing codes *or* existent local registries of VTE patients. The billing codes used were:

1802_0322	Pulmonary Embolism
602_0320	Venous malformation (thrombosis / thromboembolic / pulmonary embolism)
731_0313	Deep Venous Thromboembolism
732_0313	Pulmonary Embolism
733_0313	Venous thromboembolism, other

Care type = 11 (meaning: new code, no follow-up)
Year start DBC = 2021

Cases were included if they were deceased or filled in the questionnaire for over 30% (including the primary exposure; SARS-CoV-2 vaccination).

Cases with questionnaire (alive)

In the questionnaire a patient was asked about the VTE. If the patient replied that no VTE had happened we checked local hospital records in more detail and corrected the data if indeed a VTE occurred.

Selection of cases with first VTE

In the primary analysis we analyses patients with a first VTE. If a participant stated in the questionnaire a VTE had happened prior to 2021 or this was registered in Dutch Statistics, this participant was excluded from the primary analysis.

3-Selection of True Controls

Excluding VTE

Controls reporting VTE in 2021 were excluded. Controls with a registration of VTE in Dutch Statistics were excluded.

Excluding low quality

If a control filled in a questionnaire within 3 minutes and this questionnaire had only negative responses (quickest answer), this control was excluded.

4-Definition VTE Risk factor

The definition used is based, but no exact conform, the proposed methodology of the Guidance document of the ISTH: (https://www.sciencedirect.com/science/article/pii/S1538783622046591?via%3Dihub).

- Risk factors can be persistent of transient and major or minor.
  - Persistent
    - Major: active cancer
    - Minor: >2 fold increased risk
  - Transient
    - Major: >10 fold increased risk
    - Minor: >3 but <10 fold increased risk

The following timing rules were used, conform the Guidance document of the ISTH:

- A persistent factor should be registered or reported before the index date.
- A **major** transient factor should be registered or reported **90 days** before the index date
- A **minor** transient factor should be registered or reported **60 days** before the index date

The following factors were used for the definition of a VTE risk factor

Persistent major	Persistent minor	Transient major	Transient minor
○ Active cancer (<5 years before index date)	○ History of cancer (>5 years before index date)<br>○ Inflammatory bowel disease<br>○ Paralysis<br>○ Asthma<br>○ Multiple Sclerosis<br>○ Rheumatoid Arthritis	○ Surgery<br>○ Immobilization >3 days	○ Injury<br>○ *Cast wearing*<br>○ Estrogen use<br>○ Pregnancy<br>○ *Traveling (>4 hours)*<br>○ Infection<br>○ Arterial cardiovascular events

Apart from *Cast wearing* and *Traveling*, all risk factors could be derived both from data from Statistic Netherlands as from the questionnaire. In case of discrepancy between both data sources we used the following approach. In case the risk factor would be a definitive reason to seek healthcare (e.g. cancer, surgery) we considered the risk factor present if the participant indicated this in the questionnaire OR if it was registered in at least 2 of the 3 data sources used in Statistics Netherlands (Medication (ATC4), Billing codes (DBC) or diagnostic codes (ICD10)).

5-Used Codes in Dutch Statistics

Within Dutch Statistics we used multiple datasets containing medical information. This medical information could be codes using ICD10 codes or billing codes (DBC). A healthcare provider codes a diagnosis and treatment combination (DBC) for reimbursement from health insurance. Medication was codes using ATC4.

For VTE we used the following codes:

ICD 10 codes used for the definition of VTE

- i.801: Phlebitis and thrombophlebitis of femoral vein
- i.802: Phlebitis and thrombophlebitis of other deep vessels of lower extremities
- i.803: Phlebitis and thrombophlebitis of lower extremities, unspecified
- i.808: Phlebitis and thrombophlebitis of other sites
- i.809: Phlebitis and thrombophlebitis of unspecified site
- i.81: Portal vein thrombosis
- i.822: Embolism and thrombosis of vena cava
- i.823: Embolism and thrombosis of renal vein
- i.828: Embolism and thrombosis of other specified veins
- i.829: Embolism and thrombosis of unspecified vein
- o.223: Deep phlebothrombosis in pregnancy
- o.871: Deep phlebothrombosis in the puerperium
- o.873: Cerebral venous thrombosis in the puerperium
- o.882: Obstetric blood-clot embolism
- i.260: Pulmonary embolism with mention of acute cor pulmonale
- i.269: Pulmonary embolism without mention of acute cor pulmonale
- g.08: Intracranial and intraspinal phlebitis and thrombophlebitis
- i.676: Nonpyogenic thrombosis of intracranial venous system
- i.636: Cerebral infarction due to cerebral venous thrombosis, nonpyogenic

Billing codes used for the definition of VTE (DBC)

- 0303-03-32-0336: Mesenterial thrombosis
- 0303-04-38-0425: Deep Vein Thrombosis
- 0313-07-00-0731: Deep Vein Thrombosis
- 0313-07-00-0732: Pulmonary embolism
- 0313-07-00-0733: Deep Vein Thrombosis
- 0320-00-00-0067: Thrombosis
- 0320-06-00-0602: Thrombosis
- 0322-18-00-1802: Pulmonary embolism
- 0316-01-60-6007: Thrombosis

Examination of discrepancies between questionnaire and Dutch Statistics

If the questionnaire of a linked participants was discrepant with the data in Dutch Statistics we explored possible errors or omissions in the selection of the used codes. Using this iterative approach these selections were optimized.

For VTE risk factors we used the following codes:

Risk factor	Medication (ATC)	ICD10	DBC
Immobilization		Registration of at least 3 days in hospital	
Cancer	Antineoplastic: L01x	ICD10: (Cx)	Sub X1
Surgery		Main care activity = surgery (Sub X2)	Sub X3

6-Method of dealing with (partly) missing values in questionnaire data

In the questionnaire detailed information about VTE and VTE risk factors was asked. It was not rare participants answered yes on a certain risk factor (for example COVID-19 infection), but did not gave information about the exact date (of infection). These risk factors were analysed as complete case analysis and the effect of the missing values was assessed using two sensitivity analyses; the first assuming the risk factor as not present and the other assuming the risk factor present.

7-Description of population having a VTE in 2021

VTE occurred less often during the summer months in 2021 as compared to the fall and winter (see figure 4).

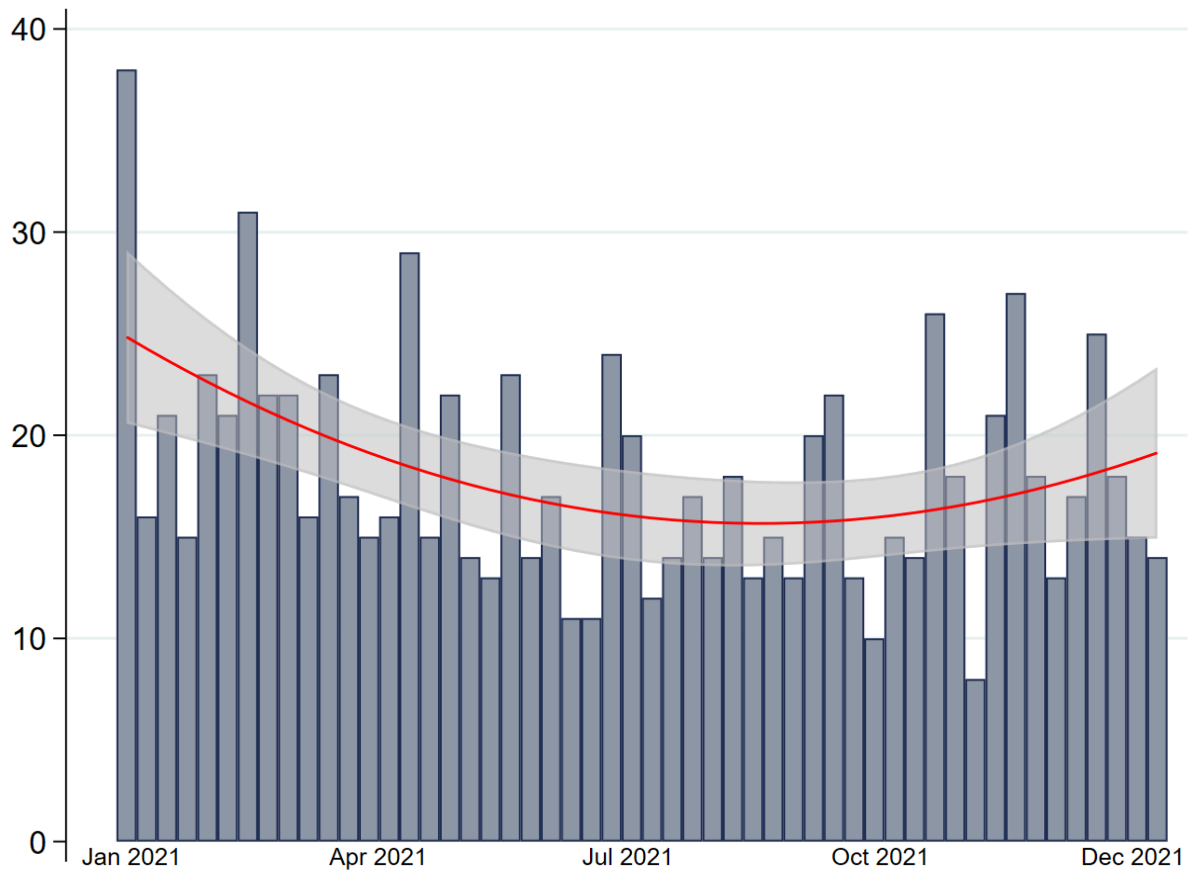

Figure: Number VTE case each week in 2021

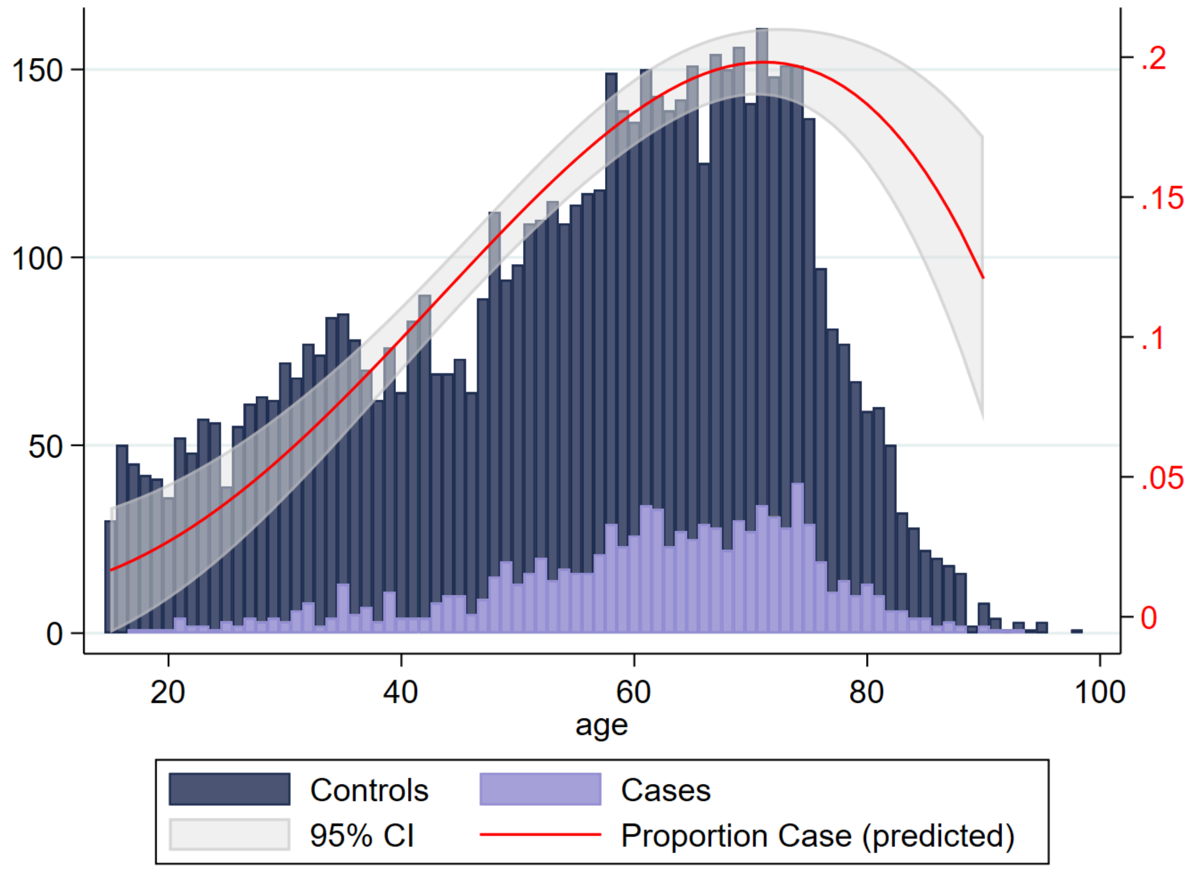

Figure: Age distribution cases compared to controls

8-Description of population vaccinated in 2021 in the Netherlands

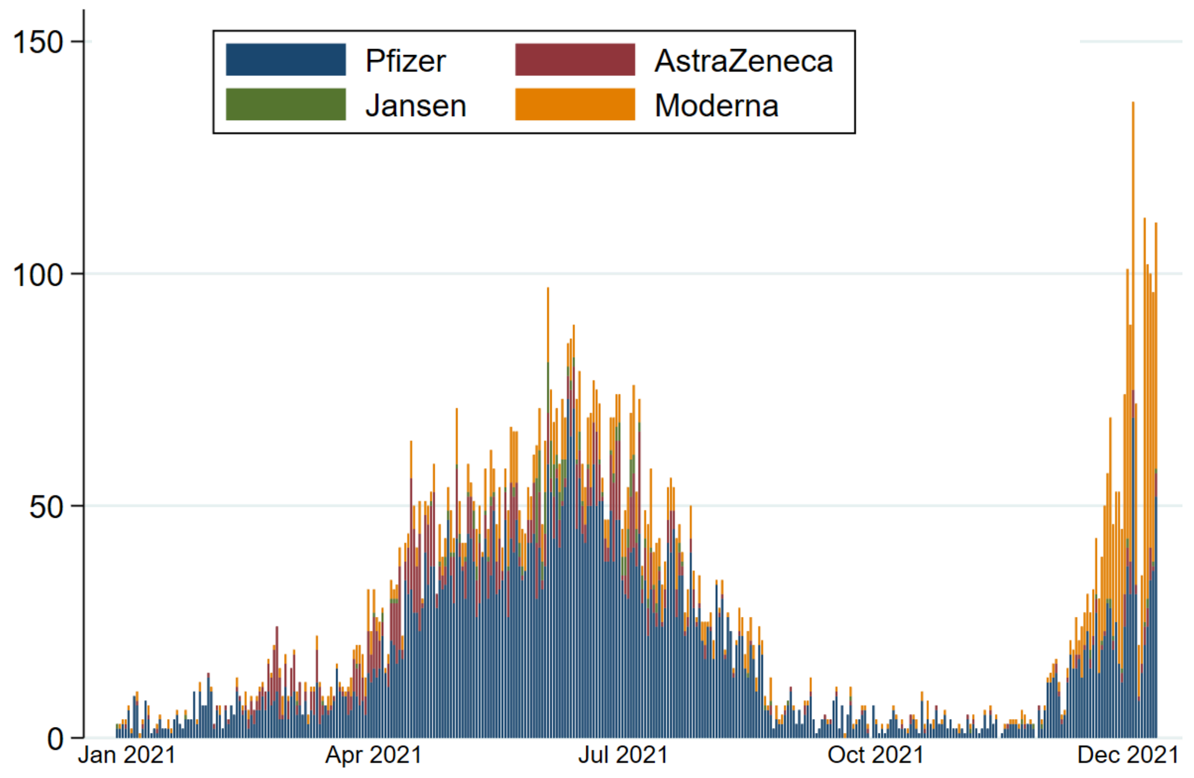

Figure: Distribution of SARS-CoV-2 vaccines over the year 2021 by type

(Jansen vaccine: Johnson&Johnson)

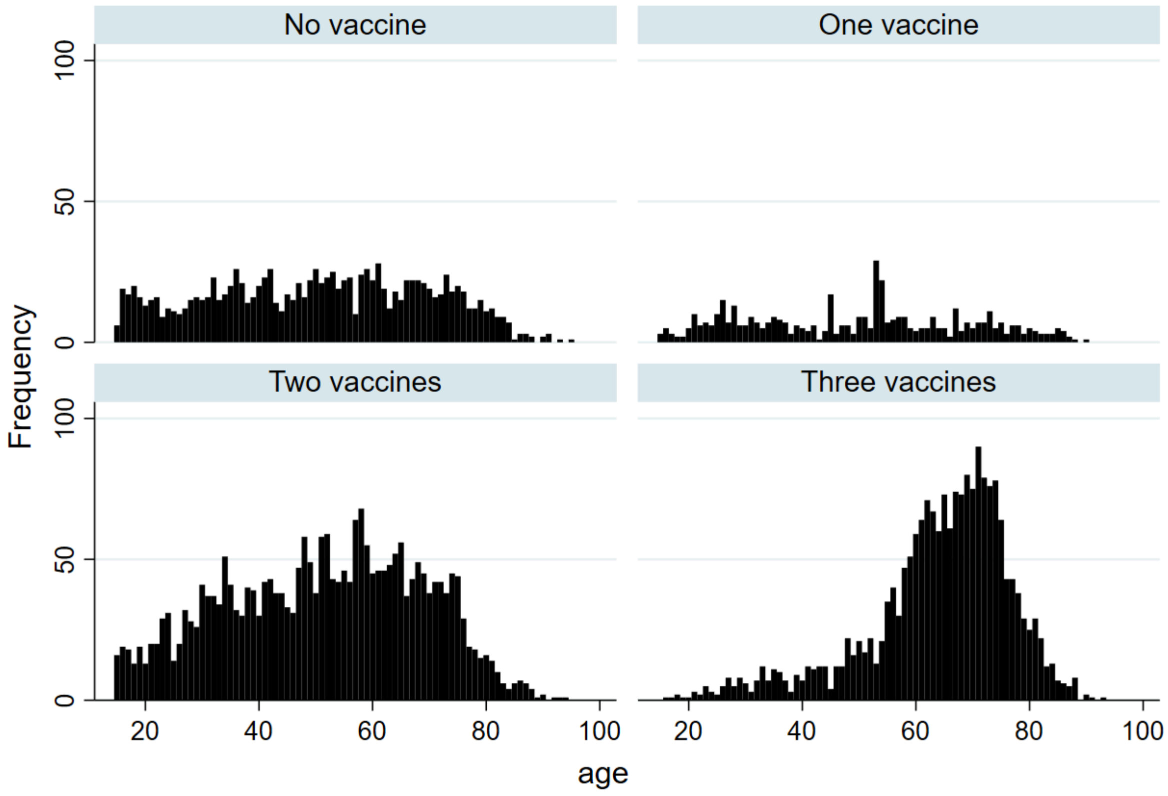

Figure: Age distribution by number of vaccines in 2021

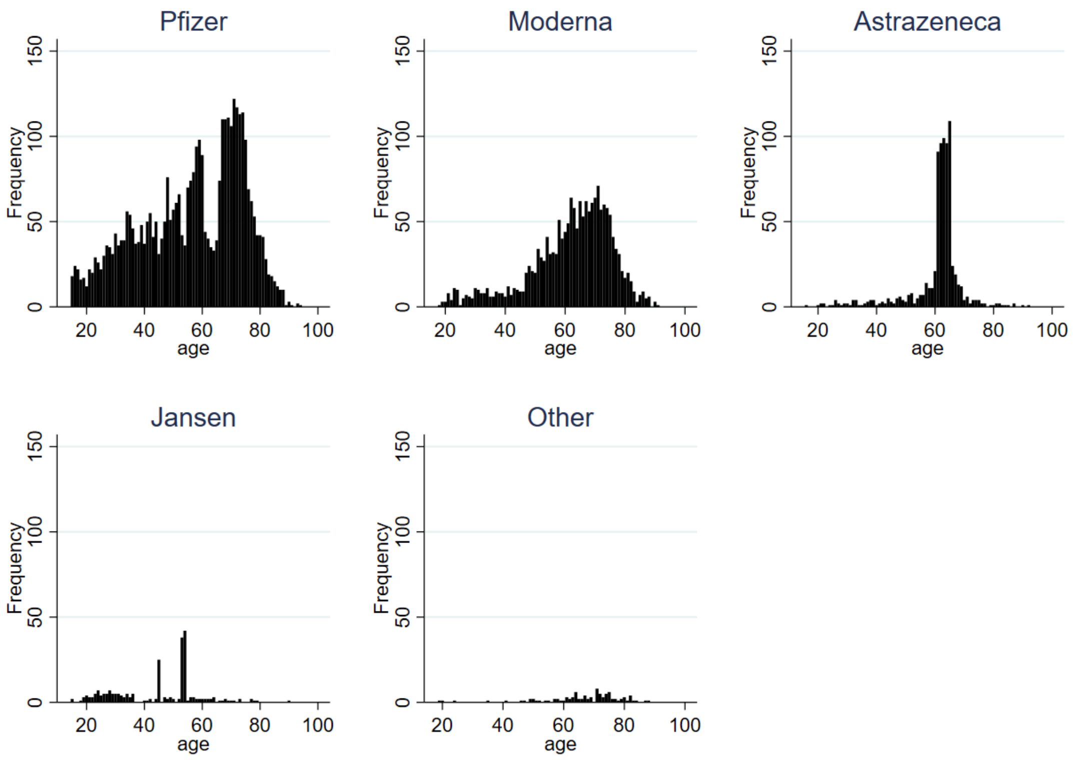

Figure: Age distribution per type of vaccin (at least one of this type)

(Jansen vaccine: Johnson&Johnson)

Codenummer

Questionnaire

TERA study

Thrombosis Etiology and Risk Assessment

Clinical Epidemiology, Leids Universitary Medical Center
Santeon Hospitals

2806027218

Guidelines for filling in the questionnaire

The questionnaire should be filled in by yourself. If some of the questions are difficult for you, you can ask someone to help you. The questions are, however, alsways about you.

Please use a black or blue pen when filling in the answers.
Most questions can be answered by filling in the circl of you choise.
Please try to stay within the circle as well as you can.

Example:

Did you undergo suergery in the last 12 months?

○ *No*

● *Yes*

If, for a certain question, more than one answer is possible, this will be stated in this question. In this case, you can fill in more than one circle.

Sometimes, we ask you to fill in a name. Please fill in these questions using capital letters.

Example

To which hospiktal were you admitted

Name

City

Sometimes we ask you about a date

example:

On what date did you receive this surgery?

day *month* *year*

Please fill in all questions. Sometimes you can skip questions, this wil than be clearly stated in the text. If a question does not concern you, please do not strike through the question but simply skip it.
If you are not sure about an answer to a question or you cannot remember a date exaclty, try and estimate this as well as you can.

If you have any questions or remarks about this study, you can write them on the last page of this questionnaire. After you filled in the questionnaire, you can send it back, together with the consent form, using the prepaid envelope.

Thank you very much for you cooperation!

7184027212

1.1. What is your year of birth

1.2. What is the gender registered in your passport?

- ○ Female
- ○ Male
- ○ X

1.3 In which country were you born?

- ○ Netherlands
- ○ Turkey
- ○ Marocco
- ○ Netherlands Antilles
- ○ Suriname
- ○ Indonesia

Other country, namely:

1.4 In which country were your father born?

- ○ Netherlands
- ○ Turkey
- ○ Marocco
- ○ Netherlands Antilles
- ○ Suriname
- ○ Indonesia

Other country, namely:

1.5 In which country were your mother born?

- ○ Netherlands
- ○ Turkey
- ○ Marocco
- ○ Netherlands Antilles
- ○ Suriname
- ○ Indonesia

Other country, namely:

1.6 What is your weight (in kilograms)?

kg

1.7 What is your height (in centimeters)?

centimeter

8481027213

2.1 Do you smoke?

○ No, I have never smoked (continue to 2.2)

○ No, but I did smoke in the past. For a total of number of years:

○ Yes, I do smoke. For in total: ☐☐ years

2.2 Do you drink alcohol?

○ No, I have never drunk alcohol

○ No, but I did drink alcohol in the past. On average:

○ Yes, I do drink alcohol. On average: ☐☐ units per week

2.3 Did you receive one or more of the following vaccines in 2021?

☐ Corona vaccine (fill in next questions)

☐ BCG (tuberculosis) vaccine, on the date: day ☐☐ month ☐☐ year ☐☐☐☐

☐ Flu shot: on the date: day ☐☐ month ☐☐ year ☐☐☐☐

☐ Other vaccine (fill in question 2.4.4)

☐ No, continue to 2.5

You can find the name and date of the vaccine on your vaccination certificate, in the coronacheck app or at https://mijn.rivm.nl/vaccinaties/login

2.4.1 Which type Corona vaccine did you receive and on what date?

○ BioNTech/ Pfizer (Comirnaty)

○ Moderna (Spikevax)

○ AstraZeneca (Vaxzevria)

○ Janssen

○ Novavax (Nuvaxovid)

day ☐☐ month ☐☐ year ☐☐☐☐

2.4.2 Which type Corona vaccine did you receive second and on what date?

(skip this question if you didn't had a second shot)

○ BioNTech/ Pfizer (Comirnaty)

○ Moderna (Spikevax)

○ AstraZeneca (Vaxzevria)

○ Janssen

○ Novavax (Nuvaxovid)

day ☐☐ month ☐☐ year ☐☐☐☐

6786027219

2.4.3 Which type Corona vaccine did you receive third and on what date?

(skip this question if you didn't had a third shot)

- ○ BioNTech/ Pfizer (Comirnaty)
- ○ Moderna (Spikevax)
- ○ AstraZeneca (Vaxzevria)
- ○ Janssen
- ○ Novavax (Nuvaxovid)

day month year

2.4.4 Which other vaccines did you receive and on what date?

day month year

day month year

day month year

day month year

day month year

8420027216

2.5 Did you exercise in 2021?

- ○ No, continue to question 2.7
- ○ Yes, daily
- ○ Yes, 4-5 times a week
- ○ Yes, 2-3 times a week
- ○ Yes, one time a week
- ○ Yes, less than weekly

2.6. What type of sport did you do? Multiple answers are possible

☐ Athletics	☐ Dancing
☐ Badminton	☐ Tennis
☐ Fitness	☐ Soccer
☐ Gymnastics	☐ Volleybal
☐ Running	☐ Cycling
☐ Squash	☐ Swimming
☐ Hockey	☐ Basketball

Other, namely

2.7 Have you suffered an injury (e.g. sprained ankle, twisted knee of mouse arm) in 2021?

- ○ No, continue to question 2.9
- ○ Yes

2.8 What type of injury did you suffer? (multiple answers are possible)

☐ sprained wrist	☐ torn ligaments (e.g. cruciate ligaments or ankle ligaments)
☐ repetitive strain injury arm/wrist	☐ meniscus injury
☐ tennis elbow	☐ twisted ankle
☐ knee injury during soccer	
☐ twisted knee	If other:

	day	month	year
When did the first injury happen?			
When did the second injury happen?			
When did the third injury happen?			
When did the forth injury happen?			

5932027211

2.9 Have you had a plaster cast in 2021?

- ○ No, continue to 2.10
- ○ Yes

Where and when did have a plaster cast (first time)?

- ○ Leg
- ○ Arm
- ○ Other, namely:

From: day ☐☐ month ☐☐ year ☐☐☐☐

Until: day ☐☐ month ☐☐ year ☐☐☐☐

Where and when did have a plaster cast (second time)?

- ○ Leg
- ○ Arm
- ○ Other, namely:

From: day ☐☐ month ☐☐ year ☐☐☐☐

Until: day ☐☐ month ☐☐ year ☐☐☐☐

Where and when did have a plaster cast (third time)?

- ○ Leg
- ○ Arm
- ○ Other, namely:

From: day ☐☐ month ☐☐ year ☐☐☐☐

Until: day ☐☐ month ☐☐ year ☐☐☐☐

Where and when did have a plaster cast (forth time)?

- ○ Leg
- ○ Arm
- ○ Other, namely:

From: day ☐☐ month ☐☐ year ☐☐☐☐

Until: day ☐☐ month ☐☐ year ☐☐☐☐

5274027210

2.10 Did you make one more more long journeys in 2021 (wherby you spent more than 4 hours in one day in one means of transport)?

- ○ No, continue to 2.11
- ○ Yes, fill in the next questions:

On what day did you travel for the **first** time? day ☐☐ month ☐☐ Year ☐☐☐☐

What kind of transportation did you use?

- ○ Car
- ○ Train
- ○ Plane
- ○ Boat
- ○ Bus
- ○ Other, namely: ☐☐☐☐☐☐☐☐☐☐☐☐☐☐☐☐☐☐☐☐

How long was your travel?

- ○ between 0 and 4 hours
- ○ between 4 and 8 hours
- ○ between 8 and 12 hours
- ○ more than12 hours

On what day did you travel for the **second** time? day ☐☐ month ☐☐ Year ☐☐☐☐

What kind of transportation did you use?

- ○ Car
- ○ Train
- ○ Plane
- ○ Boat
- ○ Bus
- ○ Other, namely: ☐☐☐☐☐☐☐☐☐☐☐☐☐☐☐☐☐☐☐☐

How long was your travel?

- ○ between 0 and 4 hours
- ○ between 4 and 8 hours
- ○ between 8 and 12 hours
- ○ more than12 hours

On what day did you travel for the **third** time? day ☐☐ month ☐☐ Year ☐☐☐☐

What kind of transportation did you use?

- ○ Car
- ○ Train
- ○ Plane
- ○ Boat
- ○ Bus
- ○ Other, namely ☐☐☐☐☐☐☐☐☐☐☐☐☐☐☐☐☐☐☐☐

How long was your travel?

- ○ between 0 and 4 hours
- ○ between 4 and 8 hours
- ○ between 8 and 12 hours
- ○ more than12 hours

5732027219

On what day did you travel for the **forth** time? day | month | Year

What kind of transportation did you use?

- ○ Car
- ○ Train
- ○ Plane
- ○ Boat
- ○ Bus
- ○ Other, namely:

How long was your travel?

- ○ between 0 and 4 hours
- ○ between 4 and 8 hours
- ○ between 8 and 12 hours
- ○ more than12 hours

On what day did you travel for the **fifth** time? day | month | Year

What kind of transportation did you use?

- ○ Car
- ○ Train
- ○ Plane
- ○ Boat
- ○ Bus
- ○ Other, namely:

How long was your travel?

- ○ between 0 and 4 hours
- ○ between 4 and 8 hours
- ○ between 8 and 12 hours
- ○ more than12 hours

On what day did you travel for the **sixth** time? day | month | Year

What kind of transportation did you use?

- ○ Car
- ○ Train
- ○ Plane
- ○ Boat
- ○ Bus
- ○ Other, namely:

How long was your travel?

- ○ between 0 and 4 hours
- ○ between 4 and 8 hours
- ○ between 8 and 12 hours
- ○ more than12 hours

6342027217

2.11 Did you stay in bed for more than 4 consecutive days in 2021?

○ No, continue to 2.12

○ Yes, fill in the next questions:

The **first** time:
For how long were you in bed? From Day ☐☐ Month ☐☐ Year ☐☐☐☐ until Day ☐☐ Month ☐☐ Year ☐☐☐☐

What was the reason you were in bed? ☐☐☐☐☐☐☐☐☐☐☐☐☐☐☐☐☐☐☐☐

The **second** time:
For how long were you in bed? From Day ☐☐ Month ☐☐ Year ☐☐☐☐ until Day ☐☐ Month ☐☐ Year ☐☐☐☐

What was the reason you were in bed? ☐☐☐☐☐☐☐☐☐☐☐☐☐☐☐☐☐☐☐☐

The **third** time:
For how long were you in bed? From Day ☐☐ Month ☐☐ Year ☐☐☐☐ until Day ☐☐ Month ☐☐ Year ☐☐☐☐

What was the reason you were in bed? ☐☐☐☐☐☐☐☐☐☐☐☐☐☐☐☐☐☐☐☐

The **forth** time:
For how long were you in bed? From Day ☐☐ Month ☐☐ Year ☐☐☐☐ until Day ☐☐ Month ☐☐ Year ☐☐☐☐

What was the reason you were in bed? ☐☐☐☐☐☐☐☐☐☐☐☐☐☐☐☐☐☐☐☐

The **fifth** time:
For how long were you in bed? From Day ☐☐ Month ☐☐ Year ☐☐☐☐ until Day ☐☐ Month ☐☐ Year ☐☐☐☐

What was the reason you were in bed? ☐☐☐☐☐☐☐☐☐☐☐☐☐☐☐☐☐☐☐☐

The **sixth** time:
For how long were you in bed? From Day ☐☐ Month ☐☐ Year ☐☐☐☐ until Day ☐☐ Month ☐☐ Year ☐☐☐☐

What was the reason you were in bed? ☐☐☐☐☐☐☐☐☐☐☐☐☐☐☐☐☐☐☐☐

8607027213

3.1 Have you had a thrombosis or pulmonary embolism in 2021 or 2022?

- ○ No, continue to 3.2
- ○ Yes, fill in the next questions

The **first** thrombosis was:

- ○ Pulmonary embolism (blooth cloth in the lungs)
- ○ Right leg
- ○ Left leg
- ○ Both legs
- ○ Brain (central venous sinus thrombosis)
- ○ Other or combination, namely:

First day of complaints of this thrombosis

Day Month Year

The **second** thrombosis was:

- ○ Pulmonary embolism (blooth cloth in the lungs)
- ○ Right leg
- ○ Left leg
- ○ Both legs
- ○ Brain (central venous sinus thrombosis)
- ○ Other or combination, namely:

First day of complaints of this thrombosis

Day Month Year

The **third** thrombosis was:

- ○ Pulmonary embolism (blooth cloth in the lungs)
- ○ Right leg
- ○ Left leg
- ○ Both legs
- ○ Brain (central venous sinus thrombosis)
- ○ Other or combination, namely:

First day of complaints of this thrombosis

Day Month Year

The **forth** thrombosis was:

- ○ Pulmonary embolism (blooth cloth in the lungs)
- ○ Right leg
- ○ Left leg
- ○ Both legs
- ○ Brain (central venous sinus thrombosis)
- ○ Other or combination, namely:

First day of complaints of this thrombosis

Day Month Year

5145027217

3.2 Have you had a thrombosis or pulmonary embolism before 2021?

○ No, continue to 3.3

○ Yes, fill in the next questions:

The **first** thrombosis was:

○ Pulmonary embolism (blooth cloth in the lungs)
○ Right leg
○ Left leg
○ Both legs
○ Brain (central venous sinus thrombosis)
○ Other or combination, namely:

First day I got complaints from this thrombosis:

Day Month Year

The **second** thrombosis was:

○ Pulmonary embolism (blooth cloth in the lungs)
○ Right leg
○ Left leg
○ Both legs
○ Brain (central venous sinus thrombosis)
○ Other or combination, namely:

First day I got complaints from this thrombosis:

Day Month Year

The **third** thrombosis was:

○ Pulmonary embolism (blooth cloth in the lungs)
○ Right leg
○ Left leg
○ Both legs
○ Brain (central venous sinus thrombosis)
○ Other or combination, namely:

First day I got complaints from this thrombosis:

Day Month Year

The **forth** thrombosis was:

○ Pulmonary embolism (blooth cloth in the lungs)
○ Right leg
○ Left leg
○ Both legs
○ Brain (central venous sinus thrombosis)
○ Other or combination, namely:

First day I got complaints from this thrombosis:

Day Month Year

7390027211

3.3 Has your biological father ever had a thrombosis?

- ○ No, continue to question 3.4
- ○ I don't know, continue to question 3.4
- ○ Yes, fill in the next question:

Type of thrombosis:

- ○ Pulmonary Embolism
- ○ Thrombosis in leg
- ○ Brain (central venous sinus thrombosis)
- ○ Other/combination, namely

Years of age

Age during first thrombosis

Number of thromboses

3.4 Has your biological mother ever had a thrombosis?

- ○ No, continue to question 3.5
- ○ I don't know, continue to question 3.5
- ○ Yes, fill in the next question:

Type of thrombosis:

- ○ Pulmonary Embolism
- ○ Thrombosis in leg
- ○ Brain (central venous sinus thrombosis)
- ○ Other/combination, namely

Years of age

Age of first thrombosis

Number of thromboses

3.5 Have any of your brothers or sisters ever had thrombosis?

- ○ No, continue to 3.6
- ○ I don't know, continue to 3.6
- ○ Yes, fill in the next questions

First brother/sister:

Years of age

Age during first thrombosis

Sex ○ Female ○ Male ○ X

- ○ Pulmonary Embolism
- ○ Thrombosis in leg
- ○ Brain (central venous sinus thrombosis)
- ○ Other/combination, namely

Number of thromboses

5269027214

Second brother/sister:

Years of age

Age during first thrombosis

- O Pulmonary Embolism
- O Thrombosis in leg
- O Brain (central venous sinus thrombosis)
- O Other/combination, namely

Sex O Female O Male O X

Number of thromboses

Third brother/sister:

Years of age

Age during first thrombosis

- O Pulmonary Embolism
- O Thrombosis in leg
- O Brain (central venous sinus thrombosis)
- O Other/combination, namely

Sex O Female O Male O X

Number of thromboses

Forth brother/sister:

Years of age

Age during first thrombosis

- O Pulmonary Embolism
- O Thrombosis in leg
- O Brain (central venous sinus thrombosis)
- O Other/combination, namely

Sex O Female O Male O X

Number of thromboses

Fifth brother/sister:

Years of age

Age during first thrombosis

- O Pulmonary Embolism
- O Thrombosis in leg
- O Brain (central venous sinus thrombosis)
- O Other/combination, namely

Sex O Female O Male O X

Number of thromboses

Sixth brother/sister:

Years of age

Age during first thrombosis

- O Pulmonary Embolism
- O Thrombosis in leg
- O Brain (central venous sinus thrombosis)
- O Other/combination, namely

Sex O Female O Male O X

Number of thromboses

6811027218

3.6 Do (or did) you have biological children?

- ○ No, continue to 4.1
- ○ Yes

3.7 Did one of your children ever had thrombosis?

- ○ No, continue to 4.1
- ○ Yes, fill in the next questions:

Type thrombosis of **first** child: Age during this thrombosis: [ ][ ][ ] Years of age

- ○ Pulmonary Embolism
- ○ Thrombosis in leg
- ○ Brain (central venous sinus thrombosis)
- ○ Other/combination, namely [ ]

Sex ○ Female ○ Male ○ X

Number of thromboses [ ][ ][ ]

Type thrombosis of **second** child: Age during this thrombosis: [ ][ ][ ] Years of age

- ○ Pulmonary Embolism
- ○ Thrombosis in leg
- ○ Brain (central venous sinus thrombosis)
- ○ Other/combination, namely [ ]

Sex ○ Female ○ Male ○ X

Number of thromboses [ ][ ][ ]

Type thrombosis of **third** child: Age during this thrombosis: [ ][ ][ ] Years of age

- ○ Pulmonary Embolism
- ○ Thrombosis in leg
- ○ Brain (central venous sinus thrombosis)
- ○ Other/combination, namely [ ]

Sex ○ Female ○ Male ○ X

Number of thromboses [ ][ ][ ]

Type thrombosis of **forth** child: Age during this thrombosis: [ ][ ][ ] Years of age

- ○ Pulmonary Embolism
- ○ Thrombosis in leg
- ○ Brain (central venous sinus thrombosis)
- ○ Other/combination, namely [ ]

Sex ○ Female ○ Male ○ X

Number of thromboses [ ][ ][ ]

9354027213

Years of age

Type thrombosis of **fifth** child: Age during this thrombosis: [ ][ ][ ]

○ Pulmonary Embolism

○ Thrombosis in leg

○ Brain (central venous sinus thrombosis)

○ Other/combination, namely

Sex ○ Female ○ Male ○ X

Number of thromboses [ ][ ][ ]

Years of age

Type thrombosis of **sixth** child: Age during this thrombosis: [ ][ ][ ]

○ Pulmonary Embolism

○ Thrombosis in leg

○ Brain (central venous sinus thrombosis)

○ Other/combination, namely

Sex ○ Female ○ Male ○ X

Number of thromboses [ ][ ][ ]

4.1 Did you have one (or more) of the following conditions or chronic diseases in 2021?

- ☐ Arthrosis
- ☐ diabetes
- ☐ Heart problems (arrhythmia, heart failure)
- ☐ Kidney disease
- ☐ Rheumatism
- ☐ COPD
- ☐ Asthma
- ☐ To fast working thyroid (hyperthyroidism)
- ☐ Other, namely
- ☐ To slow working thyroid (hypothyroidism)
- ☐ Liver disease
- ☐ Peripheral arterial disease
- ☐ Varicose veins
- ☐ Paralysis
- ☐ Inflammatory bowel disease (Colitis or Crohn's disease)
- ☐ Multiple Sclerosis (MS)
- ☐ No, none of these conditions

4.2.Have you had any of the following diseases in 2021? If so, when?

	day	month	year
☐ Heart attack			
☐ Stroke, with permanent disabilities (CVA)			
☐ Stroke, with temporary disabilities (TIA)			
☐ Cerebral hemorrhage			

☐ No, none of these conditions

1881027210

Thieme

4.3 HDid you have Corona in 2020 or 2021?

- ○ No, continue to 4.4
- ○ Yes, fill in the next questions

First time Corona; When did your complaints start?

day | month | year

Where did you recover?

- ○ At home, without complaints
- ○ At home, with complaints, without extra oxygen
- ○ At home, with complaints, with extra oxygen
- ○ Hospital admission needed
- ○ Intensive Care admission needed

Second time Corona; When did your complaints start?

day | month | year

Where did you recover?

- ○ At home, without complaints
- ○ At home, with complaints, without extra oxygen
- ○ At home, with complaints, with extra oxygen
- ○ Hospital admission needed
- ○ Intensive Care admission needed

Third time Corona; When did your complaints start?

day | month | year

Where did you recover?

- ○ At home, without complaints
- ○ At home, with complaints, without extra oxygen
- ○ At home, with complaints, with extra oxygen
- ○ Hospital admission needed
- ○ Intensive Care admission needed

Forth time Corona; When did your complaints start?

day | month | year

Where did you recover?

- ○ At home, without complaints
- ○ At home, with complaints, without extra oxygen
- ○ At home, with complaints, with extra oxygen
- ○ Hospital admission needed
- ○ Intensive Care admission needed

5846027215

4.4 Did you have any other inflammation(s) or infections(s) in 2021?
(en geef de meest recente datum per doorgemaakte type infectie)

☐ Cystitis — day ☐☐ month ☐☐ year ☐☐☐☐

☐ Pneumonia (no corona) — day ☐☐ month ☐☐ year ☐☐☐☐

☐ Joint infection — day ☐☐ month ☐☐ year ☐☐☐☐

☐ Pyelonephritis (infection of the upper urinary tract) — day ☐☐ month ☐☐ year ☐☐☐☐

☐ Bursitis — day ☐☐ month ☐☐ year ☐☐☐☐

☐ Sinusitis — day ☐☐ month ☐☐ year ☐☐☐☐

☐ Jaw/tooth infection — day ☐☐ month ☐☐ year ☐☐☐☐

☐ Liver infection — day ☐☐ month ☐☐ year ☐☐☐☐

☐ Other infection, namely: ☐☐☐☐☐☐☐☐☐☐☐☐☐☐☐☐☐☐☐☐

☐ no, none of these infections — day ☐☐ month ☐☐ year ☐☐☐☐

4.5 Have you ever had cancer?

○ No, continue to 4.6

○ Yes, fill in the next questions

What type of cancer have you (had)? (Multiple answers possible)

☐ Pancreas

☐ Cervix

☐ Uterus

☐ Bone marrow (leukemia)

☐ Bladder

☐ Breast

☐ Intestine

☐ Ovaries

☐ (hodgkin) lymphoma

☐ Skin

☐ Liver

☐ Lung

☐ Stomach

☐ Kidney

☐ Eye

☐ Prostate

☐ Thyroid

☐ Esophagus

☐ Testis

Other, namely ☐☐☐☐☐☐☐☐☐☐☐☐☐☐☐

Had the cancer (or one of the forms of cancer) spread to other organs?

○ No

○ Yes

2594027212

When were you diagnosed (with your last cancer)?

day month year

What type of treatment did you receive? (multiple answers possible)

☐ No treatment (yet)

☐ Surgery

☐ Medication (chemo)

☐ Radiation

Have you recovered? (you are recovered when you no longer need to visit the specialist)

○ No

○ Yes

4.6 Have you had surgery in 2021?

○ No, continue to 4.7

○ Yes **First** surgery:

day month year

When was the surgery?

What did you have surgery on?

Second surgery:

day month year

When was the surgery?

What did you have surgery on?

Third surgery:

day month year

When was the surgery?

What did you have surgery on?

Forth surgery:

day month year

When was the surgery?

What did you have surgery on?

Fifth surgery:

day month year

When was the surgery?

What did you have surgery on?

2343027214

The next questions are for women only.
Men can continue to 5.1 Medication

4.7. Have you ever had a menstrual period?

- ○ Yes
- ○ No, continue to 4.10

4.8 When was your last menstrual period?

- ○ Less than 4 weeks ago (continue to question 4. 10)
- ○ Longer than 4 weeks ago

4.9 What is the reason your menstrual period has been longer than 4 weeks ago?

- ○ Irregular menstration
- ○ Depot contraceptive use
- ○ Menopause
- ○ Pregnancy
- ○ Surgery on uterus/ovaries
- ○ Chemo/radiotion
- ○ Continious use of birth control pill
- ○ Hormone IUD (Mirena)
- ○ Other, namely: ☐☐☐☐☐☐☐☐☐☐☐☐☐☐☐☐☐☐☐☐

4.10 Have you been pregnant in 2021?

- ○ No, continue to 4.11
- ○ Yes

How did the first pregnancy end?

- ○ Still pregnant
- ○ Liveborn child
- ○ Stillborn child
- ○ Abortion
- ○ Miscarriage
- ○ Extra Uterine pregnancy
- ○ Other, namely: ☐☐☐☐☐☐☐☐☐☐☐☐☐☐☐☐☐☐☐☐

When did this pregnancy end? (if applicable)

day ☐☐ month ☐☐ year ☐☐☐☐

How did the second pregnancy end?

- ○ Still pregnant
- ○ Liveborn child
- ○ Stillborn child
- ○ Abortion
- ○ Miscarriage
- ○ Extra Uterine pregnancy
- ○ Other, namely: ☐☐☐☐☐☐☐☐☐☐☐☐☐☐☐☐☐☐☐☐

When did this pregnancy end? (if applicable)

day ☐☐ month ☐☐ year ☐☐☐☐

4330027212

Thieme

How did the third pregnancy end?

- ○ Still pregnant
- ○ Liveborn child
- ○ Stillborn child
- ○ Abortion
- ○ Miscarriage
- ○ Extra Uterine pregnancy
- ○ Other, namely:

When did this pregnancy end? (if applicable)

day month year

How did the forth pregnancy end?

- ○ Still pregnant
- ○ Liveborn child
- ○ Stillborn child
- ○ Abortion
- ○ Miscarriage
- ○ Extra Uterine pregnancy
- ○ Other, namely:

When did this pregnancy end? (if applicable)

day month year

4.11 Did you use one (or more) of the following forms of contraception in 2021?

- ☐ Pill (please report details in next question)
- ☐ Implanon (subdermal bar), when was this inserted? day month year
- ☐ Depot contraceptive use, when was the last injection? day month year
- ☐ Hormone IUD (Mirena), When was this placed? day month year
- ☐ Other, namely
- ☐ No (continue to 5.1)

day month year

See next page about details of hormonal contraceptive pill use

7852027214

What is the name and dose found on packaging of the first birth control pill (e.g. Microgynon, 30/150 ug)

When did you use this pill? If you are still using this pill, you can fill in the date of today.

From day month year Until day month year

What is the name and dose found on packaging of the second birth control pill (e.g. Microgynon, 30/150 ug)

When did you use this pill? If you are still using this pill, you can fill in the date of today.

From day month year Until day month year

What is the name and dose found on packaging of the third birth control pill (e.g. Microgynon, 30/150 ug)

When did you use this pill? If you are still using this pill, you can fill in the date of today.

From day month year Until day month year

What is the name and dose found on packaging of the forth birth control pill (e.g. Microgynon, 30/150 ug)

When did you use this pill? If you are still using this pill, you can fill in the date of today.

From day month year Until day month year

If you take this medication for a long time, you can also fill in 01-01-2020.

If you are still taking this medication, you can fill in 01-01-2022.

1212027218

Medication

5.1 Did you use medication in 2021? (including medication not prescribed by a physician)

○ Yes

○ No, continue to the end of this questionnaire

5.2 Did you use any of the following blood thinners (anticoagulation) in 2021?

(Leave this question blank if you don't use any of this medication)

☐ Ascal / Acetylcalicylic acid / Carasalate calcium / Aspirin / Warfine

from day ☐☐ month ☐☐ year ☐☐☐☐ until day ☐☐ month ☐☐ year ☐☐☐☐

☐ Fenprocoumon (Marcoumar)

from day ☐☐ month ☐☐ year ☐☐☐☐ until day ☐☐ month ☐☐ year ☐☐☐☐

☐ Acenocoumarol (Sintrom)

from day ☐☐ month ☐☐ year ☐☐☐☐ until day ☐☐ month ☐☐ year ☐☐☐☐

☐ Apixaban(Eliquis) / Dabigatran(Pradaxa) / Edoxaban(Lixiana) / Rivaroxaban(Xarelto)

from day ☐☐ month ☐☐ year ☐☐☐☐ until day ☐☐ month ☐☐ year ☐☐☐☐

☐ Clopidogrel (Grepid/Plavix/Iscover) / Ticagrelor (Brilique) / Prasugrel (Efient)

from day ☐☐ month ☐☐ year ☐☐☐☐ until day ☐☐ month ☐☐ year ☐☐☐☐

☐ Dalteparin (fragmin) / Nadroparin (fraxiparine) / Enoxaparin

from day ☐☐ month ☐☐ year ☐☐☐☐ until day ☐☐ month ☐☐ year ☐☐☐☐

If you take this medication for a long time, you can also fill in 01-01-2020.

If you are still taking this medication, you can fill in 01-01-2022.

7562027212

5.3 Did you use hormones in 2021? For women: you do not need to mention the contraceptive pill again.

○ No

○ Yes, fill in the next questions

For example, Prednisone, an eczema cream, tablets against osteoporosis, or other kinds of hormones

Name of first hormone used:

When did you use this hormone? If you are still using this hormone you can fill in the date of today.

from day month year until day month year

Name of second hormone used:

When did you use this hormone? If you are still using this hormone you can fill in the date of today.

from day month year until day month year

Name of third hormone used:

When did you use this hormone? If you are still using this hormone you can fill in the date of today.

from day month year until day month year

Name of forth hormone used:

When did you use this hormone? If you are still using this hormone you can fill in the date of today.

from day month year until day month year

If you take this medication for a long time, you can also fill in 01-01-2020.

If you are still taking this medication, you can fill in 01-01-2022.

8211027214

Thieme

If you take this medication for a long time, you can also fill in 01-01-2020.

If you are still taking this medication, you can fill in 01-01-2022.

5.4 Did you take any other medication in 2021?

○ No

○ Ja, fill in the next questions

What medication did you use?

When did you use this medication? If you still use this medication you can fill in the date of today.

from day month year until day month year

What medication did you use?

When did you use this medication? If you still use this medication you can fill in the date of today.

from day month year until day month year

What medication did you use?

When did you use this medication? If you still use this medication you can fill in the date of today.

from day month year until day month year

What medication did you use?

When did you use this medication? If you still use this medication you can fill in the date of today.

from day month year until day month year

What medication did you use?

When did you use this medication? If you still use this medication you can fill in the date of today.

from day month year until day month year

What medication did you use?

When did you use this medication? If you still use this medication you can fill in the date of today.

from day month year until day month year

7819027217

What medication did you use?

When did you use this medication? If you still use this medication you can fill in the date of today.

from day month year until day month year

What medication did you use?

When did you use this medication? If you still use this medication you can fill in the date of today.

from day month year until day month year

What medication did you use?

When did you use this medication? If you still use this medication you can fill in the date of today.

from day month year until day month year

What medication did you use?

When did you use this medication? If you still use this medication you can fill in the date of today.

from day month year until day month year

Thank you very much for completing this questionnaire.
Please send the questionnaire together with the consent form back to us, using the prepaid envelope.

If you have any additional remarks about the questionnaire or this study, you can write it down below.

3833027219

					mRNA						Vector					
					BNT162b2 (Pfizer)			mRNA-1273 (Moderna)			AZD1222 (AstraZeneca)			Ad26.COV2.S (Johnson & Johnson)		
			Cases	Controls	aOR	95% CI		aOR	95% CI		aOR	95% CI		aOR	95% CI	
Any VTE	**Any dose**	Total	779	5311	1,0	0,7	1,3	1,4	0,8	2,4	1,5	1,0	2,5	2,9	0,9	9,2
Any VTE	**Any dose**	male	438	2476	0,8	0,6	1,2	1,7	0,9	3,3	1,3	0,7	2,6	4,4	1,1	18,1
Any VTE	**Any dose**	female	341	2835	1,2	0,8	1,7	1,1	0,5	2,5	1,8	0,9	3,7	1,6	0,2	13,7
Any VTE	**Any dose**	<=60	327	3118	1,1	0,7	1,6	1,0	0,4	2,6	2,0	0,7	5,7	3,5	1,1	11,4
Any VTE	**Any dose**	>60	452	2193	0,9	0,6	1,4	1,5	0,8	2,9	1,2	0,7	2,1			
Any VTE	**First vaccine**	Total	779	5311	1,1	0,8	1,6	2,4	1,1	5,5	1,8	1,0	3,3	3,0	0,9	9,3
Any VTE	**First vaccine**	male	438	2476	1,0	0,6	1,7	3,4	1,2	9,4	1,4	0,6	3,3	4,5	1,1	18,7
Any VTE	**First vaccine**	female	341	2835	1,2	0,7	2,0	1,3	0,3	5,8	2,3	1,0	5,4			
Any VTE	**First vaccine**	<=60	327	3118	1,2	0,7	2,2	0,9	0,2	4,0	3,0	0,9	10,4	3,5	1,1	11,6
Any VTE	**First vaccine**	>60	452	2193	1,1	0,7	1,8	5,4	1,7	17,3	1,3	0,6	2,6			
Any VTE	**Second vaccine**	Total	779	5311	0,9	0,6	1,3	1,5	0,6	4,1	1,2	0,5	2,0			
Any VTE	**Second vaccine**	male	438	2476	0,7	0,4	1,2	2,7	0,8	8,8	1,1	0,4	2,2			
Any VTE	**Second vaccine**	female	341	2835	1,1	0,6	1,8	0,6	0,1	4,6	1,2	0,4	2,5			
Any VTE	**Second vaccine**	<=60	327	3118	0,9	0,5	1,6	1,2	0,3	4,1						
Any VTE	**Second vaccine**	>60	452	2193	0,9	0,5	1,5	1,8	0,3	9,0	1,0	0,4	1,9			
PE	**Any dose**	Total	422	5311	0,9	0,6	1,3	1,4	0,7	2,6	1,3	0,7	2,5	3,8	1,1	14,0
PE	**Any dose**	male	247	2476	0,9	0,5	1,4	1,2	0,5	3,1	1,3	0,6	3,1	8,3	2,0	34,9
PE	**Any dose**	female	175	2835	0,9	0,5	1,6	1,5	0,6	4,0	1,2	0,4	3,6			
PE	**Any dose**	<=60	171	3118	1,2	0,7	2,2	0,7	0,2	3,1	0,7	0,1	5,3	4,1	1,1	15,7
PE	**Any dose**	>60	251	2193	0,8	0,5	1,3	1,6	0,7	3,7	1,3	0,6	2,7			
PE	**First vaccine**	Total	422	5311	1,1	0,7	1,7	2,9	1,1	7,8	1,7	0,8	3,7	4,0	1,1	14,7
PE	**First vaccine**	male	247	2476	1,0	0,6	2,0	3,1	0,8	11,6	1,6	0,6	4,5	8,6	2,1	36,2
PE	**First vaccine**	female	175	2835	1,1	0,5	2,2	2,5	0,5	11,4	1,7	0,5	6,0			
PE	**First vaccine**	<=60	171	3118	1,2	0,6	2,5	0,8	0,1	6,0	1,2	0,1	10,0	4,0	1,1	15,3
PE	**First vaccine**	>60	251	2193	1,1	0,5	2,1	7,5	2,0	28,2	1,7	0,7	4,1			
PE	**Second vaccine**	Total	422	5311	0,8	0,5	1,4	1,2	0,3	5,1	0,9	0,3	3,0			
PE	**Second vaccine**	male	247	2476	0,9	0,5	1,7	1,2	0,2	9,9	1,0	0,2	4,3			
PE	**Second vaccine**	female	175	2835	0,7	0,3	1,7	1,2	0,2	9,5	0,8	0,1	5,8			
PE	**Second vaccine**	<=60	171	3118	1,2	0,6	2,6	0,8	0,1	6,1						
PE	**Second vaccine**	>60	251	2193	0,8	0,4	1,7	1,9	0,2	15,9	1,0	0,3	3,6			
DVT	**Any dose**	Total	273	5311	1,1	0,7	1,7	1,6	0,7	3,6	1,8	0,9	3,7	2,1	0,3	16,3
DVT	**Any dose**	male	157	2476	0,8	0,4	1,5	2,9	1,1	7,4	1,5	0,6	3,9			
DVT	**Any dose**	female	116	2835	1,6	0,9	2,8	0,5	0,1	3,4	2,3	0,8	6,8	5,8	0,7	50,1
DVT	**Any dose**	<=60	114	3118	0,8	0,4	1,7	1,8	0,5	6,0	4,1	1,1	15,1	2,7	0,3	22,6
DVT	**Any dose**	>60	159	2193	1,4	0,8	2,4	1,5	0,5	4,5	1,0	0,4	2,4			
DVT	**First vaccine**	Total	273	5311	1,0	0,6	1,9	2,7	0,8	9,3	1,8	0,7	4,6	1,9	0,2	15,3
DVT	**First vaccine**	male	157	2476	0,8	0,4	1,9	4,6	1,2	17,7	1,0	0,2	4,6			
DVT	**First vaccine**	female	116	2835	1,4	0,6	3,3				2,9	0,8	10,8	5,2	0,6	45,2
DVT	**First vaccine**	<=60	114	3118	0,6	0,2	2,2	1,5	0,2	12,0	4,9	1,0	25,2	2,4	0,3	20,6
DVT	**First vaccine**	>60	159	2193	1,4	0,7	2,8	5,2	1,0	27,7	0,8	0,2	2,9			
DVT	**Second vaccine**	Total	273	5311	1,0	0,6	1,8	2,6	0,8	8,8	1,4	0,4	4,6			
DVT	**Second vaccine**	male	157	2476	0,6	0,2	1,5	6,0	1,6	22,8	1,4	0,3	6,3			
DVT	**Second vaccine**	female	116	2835	1,6	0,8	3,4				1,3	0,2	10,1			
DVT	**Second vaccine**	<=60	114	3118	1,0	0,4	2,5	2,2	0,5	9,7	3,0	0,4	26,2			
DVT	**Second vaccine**	>60	159	2193	1,0	0,5	2,3	2,3	0,3	20,2	0,8	0,2	3,5			
OTHER	**Any dose**	Total	84	5311	0,8	0,4	1,8	1,4	0,3	6,1	2,1	0,6	7,1			

Supplementary Table 1A: Relative risks (Odds Ratios) for several SARS-CoV-2 vaccines in several groups for different outcomes within 28 days

VTE: Venous thromboembolism; PE: Pulmonary embolism. DVT: Deep vein thrombosis. aOR: adjusted Odds ratio (Adjusted for age, sex, BMI, calendar time (month))

					mRNA						Vector					
					BNT162b2 (Pfizer)			mRNA-1273 (Moderna)			AZD1222 (AstraZeneca)			Ad26.COV2.S (Johnson & Johnson)		
			Cases	Controls	aOR	95% CI		aOR	95% CI		aOR	95% CI		aOR	95% CI	
Any VTE	**Any dose**	Total	779	5311	0,9	0,7	1,3	1,4	0,8	2,6	1,8	1,0	3,3	3,8	1,0	15,3
Any VTE	**Any dose**	male	438	2476	0,8	0,5	1,3	1,5	0,7	3,3	2,0	0,9	4,4	4,6	0,8	26,3
Any VTE	**Any dose**	female	341	2835	1,1	0,7	1,8	1,4	0,6	3,5	1,6	0,7	3,9	3,5	0,4	35,0
Any VTE	**Any dose**	<=60	327	3118	1,1	0,6	1,9	1,5	0,5	4,4	2,9	0,8	9,7	5,6	1,3	23,7
Any VTE	**Any dose**	>60	452	2193	0,9	0,5	1,4	1,3	0,6	2,7	1,3	0,7	2,6			
Any VTE	**First vaccine**	Total	779	5311	1,1	0,7	1,8	2,4	0,9	7,0	2,0	0,9	4,3	3,9	1,0	15,4
Any VTE	**First vaccine**	male	438	2476	1,2	0,6	2,2	3,9	1,0	14,7	2,2	0,7	6,6	4,7	0,8	27,0
Any VTE	**First vaccine**	female	341	2835	1,1	0,5	2,3	1,0	0,1	7,9	1,9	0,6	5,4	3,5	0,4	34,6
Any VTE	**First vaccine**	<=60	327	3118	1,1	0,5	2,5	0,7	0,1	5,5	4,3	0,9	19,7	5,5	1,3	23,0
Any VTE	**First vaccine**	>60	452	2193	1,2	0,6	2,4	6,6	1,4	30,9	1,4	0,6	3,4			
Any VTE	**Second vaccine**	Total	779	5311	0,8	0,5	1,4	2,4	0,8	7,3	1,7	0,7	2,6			
Any VTE	**Second vaccine**	male	438	2476	0,6	0,3	1,3				2,0	0,6	3,1			
Any VTE	**Second vaccine**	female	341	2835	1,1	0,5	2,2	1,4	0,2	11,1						
Any VTE	**Second vaccine**	<=60	327	3118	1,1	0,5	2,2	3,1	0,8	11,8						
Any VTE	**Second vaccine**	>60	452	2193	0,6	0,3	1,4	1,0	0,1	9,0	1,4	0,5	2,4			
PE	**Any dose**	Total	422	5311	1,0	0,6	1,6	1,3	0,6	2,9	1,4	0,6	3,2	4,5	0,9	22,3
PE	**Any dose**	male	247	2476	1,0	0,5	1,8	1,0	0,3	3,0	2,3	0,9	5,9	8,8	1,5	51,1
PE	**Any dose**	female	175	2835	1,0	0,5	2,0	1,8	0,6	5,4	0,4	0,1	3,4			
PE	**Any dose**	<=60	171	3118	1,3	0,7	2,6	1,3	0,3	5,7	1,2	0,1	10,0	5,7	1,1	30,5
PE	**Any dose**	>60	251	2193	0,9	0,5	1,7	1,2	0,5	3,2	1,3	0,5	3,3			
PE	**First vaccine**	Total	422	5311	1,1	0,6	2,1	3,7	1,2	11,6	1,8	0,7	4,9	4,5	0,9	22,6
PE	**First vaccine**	male	247	2476	1,4	0,6	3,0	5,3	1,2	22,5	3,0	0,9	10,0	8,8	1,5	51,1
PE	**First vaccine**	female	175	2835	0,7	0,2	2,2	1,8	0,2	14,8	0,7	0,1	5,6			
PE	**First vaccine**	<=60	171	3118	1,1	0,4	3,0	1,2	0,1	9,6	2,3	0,2	21,6	5,4	1,0	29,1
PE	**First vaccine**	>60	251	2193	1,1	0,4	2,6	9,7	1,8	51,2	1,6	0,5	4,9			
PE	**Second vaccine**	Total	422	5311	1,0	0,5	1,9	1,1	0,1	8,9	1,0	0,2	4,4			
PE	**Second vaccine**	male	247	2476	0,9	0,4	2,1				1,7	0,4	7,9			
PE	**Second vaccine**	female	175	2835	1,2	0,5	3,1	2,9	0,3	23,9						
PE	**Second vaccine**	<=60	171	3118	1,4	0,6	3,5	2,1	0,3	17,1						
PE	**Second vaccine**	>60	251	2193	0,9	0,3	2,3				1,2	0,3	5,2			
DVT	**Any dose**	Total	273	5311	0,8	0,5	1,5	1,5	0,6	3,9	2,3	1,0	5,3	3,4	0,4	28,7
DVT	**Any dose**	male	157	2476	0,5	0,2	1,3	2,4	0,8	7,6	1,7	0,5	5,7			
DVT	**Any dose**	female	116	2835	1,3	0,6	2,9	0,6	0,1	4,9	3,2	1,0	9,8	12,1	1,2	124,7
DVT	**Any dose**	<=60	114	3118	0,7	0,3	2,1	2,4	0,5	10,8	8,0	2,0	32,1	6,4	0,7	58,8
DVT	**Any dose**	>60	159	2193	0,9	0,4	1,9	1,2	0,3	4,3	1,1	0,4	3,1			
DVT	**First vaccine**	Total	273	5311	1,1	0,5	2,4	1,5	0,2	12,0	2,5	0,9	7,6	3,4	0,4	29,0
DVT	**First vaccine**	male	157	2476	0,7	0,2	2,5	2,8	0,3	24,7	1,2	0,1	9,2			
DVT	**First vaccine**	female	116	2835	1,8	0,6	5,2				4,3	1,1	16,4	12,3	1,2	126,6
DVT	**First vaccine**	<=60	114	3118	0,4	0,1	3,4				9,8	1,6	58,4	5,5	0,6	51,2
DVT	**First vaccine**	>60	159	2193	1,6	0,6	4,0	5,2	0,5	53,8	1,0	0,2	4,7			
DVT	**Second vaccine**	Total	273	5311	0,6	0,3	1,6	4,7	1,3	16,9	2,2	0,6	7,5			
DVT	**Second vaccine**	male	157	2476	0,4	0,1	1,8	9,3	2,2	38,8	2,4	0,5	11,3			
DVT	**Second vaccine**	female	116	2835	1,0	0,3	3,1				1,8	0,2	14,3			
DVT	**Second vaccine**	<=60	114	3118	1,0	0,3	3,4	6,1	1,2	29,5	5,6	0,6	52,8			
DVT	**Second vaccine**	>60	159	2193	0,4	0,1	1,6	2,6	0,3	22,9	1,2	0,3	5,6			
OTHER	**Any dose**	Total	84	5311	0,9	0,3	2,6	2,0	0,4	9,0	2,3	0,5	10,3			

Supplementary Table 1B: Relative risks (Odds Ratios) for several SARS-CoV-2 vaccines in several groups for different outcomes within 14 days

VTE: Venous thromboembolism; PE: Pulmonary embolism. DVT: Deep vein thrombosis. aOR: adjusted Odds ratio (Adjusted for age, sex, BMI, calendar time (month))

Thieme

					mRNA						Vector					
					BNT162b2 (Pfizer)			mRNA-1273 (Moderna)			AZD1222 (AstraZeneca)			Ad26.COV2.S (Johnson & Johnson)		
			**Cases**	**Controls**	**aOR**	**95% CI**		**aOR**	**95% CI**		**aOR**	**95% CI**		**aOR**	**95% CI**	
Any VTE	**Any dose**	Total	779	5311	0,9	0,8	1,2	1,3	0,9	1,9	1,0	0,7	1,3	1,6	0,9	2,9
Any VTE	**Any dose**	male	438	2476	1,0	0,7	1,3	1,6	1,0	2,6	1,1	0,7	1,7	2,1	1,0	4,4
Any VTE	**Any dose**	female	341	2835	0,9	0,7	1,2	1,1	0,6	1,8	0,8	0,5	1,3	1,2	0,5	3,2
Any VTE	**Any dose**	<=60	327	3118	0,9	0,6	1,2	1,1	0,6	1,8	0,9	0,5	1,8	1,6	0,8	2,9
Any VTE	**Any dose**	>60	452	2193	1,0	0,7	1,4	1,4	0,8	2,3	0,8	0,5	1,1			
Any VTE	**First vaccine**	Total	779	5311	1,2	0,9	1,6	1,8	1,0	3,4	1,1	0,7	1,7	1,7	0,9	2,9
Any VTE	**First vaccine**	male	438	2476	1,2	0,8	1,8	2,2	1,0	4,7	1,1	0,6	2,0	2,1	1,0	4,3
Any VTE	**First vaccine**	female	341	2835	1,1	0,7	1,7	1,4	0,5	4,1	1,1	0,5	2,1	1,3	0,5	3,4
Any VTE	**First vaccine**	<=60	327	3118	1,2	0,8	1,9	1,0	0,4	2,7	2,4	1,0	5,6	1,7	1,0	3,1
Any VTE	**First vaccine**	>60	452	2193	1,2	0,8	1,8	3,3	1,3	8,2	0,7	0,4	1,2			
Any VTE	**Second vaccine**	Total	779	5311	0,8	0,7	1,0	1,3	0,8	2,0	0,8	0,6	1,2			
Any VTE	**Second vaccine**	male	438	2476	0,8	0,6	1,1	1,6	0,9	3,0	0,9	0,6	1,4			
Any VTE	**Second vaccine**	female	341	2835	0,8	0,6	1,2	1,0	0,5	2,0	0,7	0,4	1,3			
Any VTE	**Second vaccine**	<=60	327	3118	0,7	0,5	1,0	1,0	0,6	1,8	0,4	0,2	1,4			
Any VTE	**Second vaccine**	>60	452	2193	1,0	0,7	1,4	1,6	0,7	3,5	0,8	0,5	1,3			
PE	**Any dose**	Total	422	5311	0,8	0,6	1,1	1,2	0,7	1,9	0,9	0,6	1,3	1,4	0,6	3,1
PE	**Any dose**	male	247	2476	0,9	0,6	1,4	1,3	0,7	2,5	1,1	0,7	1,9	2,0	0,7	5,4
PE	**Any dose**	female	175	2835	0,6	0,4	1,0	1,0	0,5	2,0	0,6	0,3	1,2	0,9	0,2	3,8
PE	**Any dose**	<=60	171	3118	0,8	0,5	1,3	0,8	0,4	1,8	0,7	0,3	1,8	1,4	0,6	3,3
PE	**Any dose**	>60	251	2193	0,8	0,5	1,3	1,5	0,8	2,9	0,8	0,5	1,3			
PE	**First vaccine**	Total	422	5311	1,0	0,7	1,6	2,2	1,0	4,8	1,3	0,8	2,3	1,6	0,7	3,5
PE	**First vaccine**	male	247	2476	1,1	0,7	1,9	1,8	0,6	5,3	1,6	0,8	3,2	2,0	0,8	5,4
PE	**First vaccine**	female	175	2835	0,9	0,5	1,7	2,8	0,9	8,2	1,0	0,4	2,5	1,2	0,3	5,0
PE	**First vaccine**	<=60	171	3118	1,2	0,6	2,1	1,2	0,3	3,9	2,1	0,7	6,2	1,7	0,7	3,8
PE	**First vaccine**	>60	251	2193	0,9	0,5	1,7	4,3	1,5	12,8	1,1	0,5	2,1			
PE	**Second vaccine**	Total	422	5311	0,8	0,6	1,1	1,1	0,6	2,1	0,7	0,4	1,2			
PE	**Second vaccine**	male	247	2476	0,9	0,6	1,3	1,5	0,6	3,5	0,9	0,4	1,7			
PE	**Second vaccine**	female	175	2835	0,6	0,4	1,0	0,8	0,3	2,2	0,5	0,2	1,3			
PE	**Second vaccine**	<=60	171	3118	0,7	0,4	1,1	0,7	0,3	1,7	0,2	0,0	1,2			
PE	**Second vaccine**	>60	251	2193	1,0	0,7	1,6	2,2	0,8	5,6	0,8	0,4	1,5			
DVT	**Any dose**	Total	273	5311	1,3	0,9	1,9	1,9	1,1	3,4	1,4	0,8	2,2	2,9	1,3	6,4
DVT	**Any dose**	male	157	2476	1,2	0,7	1,9	2,4	1,1	5,1	1,5	0,8	2,8	3,4	1,2	9,7
DVT	**Any dose**	female	116	2835	1,5	0,9	2,6	1,6	0,7	3,8	1,2	0,6	2,6	2,6	0,7	9,2
DVT	**Any dose**	<=60	114	3118	0,9	0,5	1,7	1,7	0,8	3,7	1,5	0,6	3,7	2,5	1,0	6,0
DVT	**Any dose**	>60	159	2193	1,7	1,0	2,9	1,6	0,6	3,8	0,9	0,5	1,7			
DVT	**First vaccine**	Total	273	5311	1,2	0,8	2,0	1,6	0,6	4,6	0,8	0,4	1,8	2,3	1,1	4,9
DVT	**First vaccine**	male	157	2476	1,1	0,6	2,1	2,7	0,9	8,0	0,5	0,2	1,8	2,8	1,0	7,4
DVT	**First vaccine**	female	116	2835	1,4	0,7	2,7				1,2	0,4	3,6	2,0	0,6	6,6
DVT	**First vaccine**	<=60	114	3118	0,6	0,2	1,6	0,6	0,1	4,4	2,7	0,8	9,8	2,3	1,0	4,9
DVT	**First vaccine**	>60	159	2193	1,8	1,0	3,4	3,2	0,9	11,9	0,3	0,1	1,0			
DVT	**Second vaccine**	Total	273	5311	1,0	0,7	1,4	2,1	1,1	3,9	1,2	0,7	2,0			
DVT	**Second vaccine**	male	157	2476	0,9	0,6	1,4	2,6	1,1	6,2	1,5	0,8	2,9			
DVT	**Second vaccine**	female	116	2835	1,2	0,7	1,9	1,8	0,8	4,4	0,7	0,3	2,0			
DVT	**Second vaccine**	<=60	114	3118	0,9	0,5	1,6	2,0	0,9	4,2	1,0	0,3	3,1			
DVT	**Second vaccine**	>60	159	2193	1,1	0,7	1,8	1,4	0,4	5,1	0,9	0,5	1,8			
OTHER	**Any dose**	Total	84	5311	0,8	0,4	1,4	0,8	0,3	2,3	0,5	0,2	1,4			

Supplementary Table 1C: Relative risks (Odds Ratios) for several SARS-CoV-2 vaccines in several groups for different outcomes within 180 days

VTE: Venous thromboembolism; PE: Pulmonary embolism. DVT: Deep vein thrombosis. aOR: adjusted Odds ratio (Adjusted for age, sex, BMI, calendar time (month))

Supplementary table 2: Odds ratios describing association between VTE, VTE risk factors and vaccines in the past 28 days

Risk factor	Vaccination	BNT162b2 (Pfizer-BioNTech)	mRNA-1273 (Moderna)	AZD1222 (AstraZeneca)	Ad26.COV2.S (Johnson & Johnson)
**Any**					
-	-	1 [ref]	1 [ref]	1 [ref]	1 [ref]
+	-	10.8 (8.8 – 13.2)	10.8 (8.8 – 13.2)	10.8 (8.8 – 13.2)	10.8 (8.8 – 13.2)
-	+	1.5 (1.1 – 2.1)	0.8 (0.3 – 2.3)	2.0 (1.0 – 3.9)	3.4 (0.7 – 15.5)
+	+	6.2 (3.7 – 10.5)	15.1 (6.5 – 34.9)	12.1 (4.8 – 30.2)	35.0 (3.0 – 1414.6)
**Cancer**					
-	-	1 [ref]	1 [ref]	1 [ref]	1 [ref]
+	-	3.9 (2.9 – 5.3)	3.9 (2.9 – 5.3)	3.9 (2.9 – 5.3)	3.9 (2.9 – 5.3)
-	+	1.1 (0.8 – 1.5)	1.2 (0.6 – 2.2)	1.6 (0.9 – 2.7)	3.3 (1.1 – 10.5)
+	+	2.2 (1.0 – 4.5)	5.3 (1.6 – 17.2)	4.8 (1.1 – 20.6)	-
**Immobilization**					
-	-	1 [ref]	1 [ref]	1 [ref]	1 [ref]
+	-	25.6 (18.9 – 34.5)	25.6 (18.9 – 34.5)	25.6 (18.9 – 34.5)	25.6 (18.9 – 34.5)
-	+	1.2 (0.9 – 1.6)	1.9 (1.0 – 3.5)	1.8 (1.0 – 3.2)	2.6 (0.6 – 11.9)
+	+	13.9 (6.3 – 30.3)	18.6 (3.2 – 107.1)	19.1 (4.5 – 81.2)	31.4 (2.6 – 383.1)
**Surgery**					
-	-	1 [ref]	1 [ref]	1 [ref]	1 [ref]
+	-	8.4 (6.2 – 11.4)	8.4 (6.2 – 11.4)	8.4 (6.2 – 11.4)	8.4 (6.2 – 11.4)
-	+	1.1 (0.8 – 1.5)	1.3 (0.7 – 2.4)	2.0 (1.2 – 3.3)	4.3 (1.3 – 13.5)
+	+	5.9 (2.3 – 15.2)	8.2 (1.9 – 34.9)	-	-
**COVID-19**					
-	-	1 [ref]	1 [ref]	1 [ref]	1 [ref]
+	-	6.6 (5.0 – 8.8)	6.6 (5.0 – 8.8)	6.6 (5.0 – 8.8)	6.6 (5.0 – 8.8)
-	+	1.1 (0.8 – 1.5)	1.5 (0.8 – 2.6)	1.8 (1.1 – 3.1)	3.3 (0.9 – 12.1)
+	+	6.2 (3.7 – 10.5)	15.1 (6.5 – 34.9)	12.1 (4.8 – 30.2)	35.0 (3.0 – 414.6)

* Cancer diagnosis not longer than 5 years ago, immobilization for at least 3 days in the past 90 days, surgery in the past 90 days, COVID-19 infection in the past 60 days

adjusted for age, sex and month

Distribution risk factors:

		N cases	N Controls
Cancer	+	102	194
	-	598	4979
Immobilization	+	208	98
	-	480	5091
Surgery	+	108	125
	-	579	5047
COVID-19	+	112	167
	-	568	5011
Any Riskfactor	+	349	511
	-	310	4666

				No Vaccine			mRNA						Vector					
							BNT162b2 (Pfizer)			mRNA-1273 (Moderna)			AZD1222 (AstraZeneca)			Ad26.COV2.S (Johnson & Johnson)		
		N cases	N Controls	aOR	95% CI		aOR	95% CI		aOR	95% CI		aOR	95% CI		aOR	95% CI	
Cancer	+	31	93	2,2	1,3	3,6	0,7	0,2	3,3	10,6	2,5	45,8						
	-	544	4604	REF			0,9	0,7	1,3	1,3	0,7	2,4	1,7	1,0	2,9	4,1	1,3	13,5
Immobilisation	+	161	21	81,8	48,3	138,6	35,2	11,1	111,8									
	-	414	4676	REF			1,0	0,7	1,4	2,0	1,1	3,7	1,6	0,9	3,1	4,1	1,1	15,4
Surgery	+	51	71	7,2	4,7	10,9	2,8	0,7	11,8	8,5	1,0	68,4						
	-	524	4626	REF			0,9	0,7	1,3	1,7	0,9	2,9	1,7	1,0	2,9	4,5	1,4	14,6
COVID-19	+	74	71	10,5	7,1	15,3	10,2	1,9	53,7	4,9	0,4	55,5	17,4	1,5	198,5			
	-	501	4626	REF			1,0	0,7	1,3	1,9	1,1	3,3	1,6	0,9	2,8	3,3	0,9	12,5
Any Riskfactor	+	235	243	12,2	9,6	15,5	7,0	3,8	13,1	16,6	6,0	45,4	14,1	4,2	47,5			
	-	340	4454	REF			1,1	0,7	1,6	1,5	0,7	3,2	1,8	1,0	3,4	2,7	0,6	12,8

Supplementary Table 2A: Relative risks (Odds Ratios) for the association between venous thromboembolism and several SARS-CoV-2 vaccines within 28 days in several risk groups with risk groups defined by only data from Statistics Netherlands (CBS)

				No Vaccine			mRNA						Vector					
							BNT162b2 (Pfizer)			mRNA-1273 (Moderna)			AZD1222 (AstraZeneca)			Ad26.COV2.S (Johnson & Johnson)		
		N cases	N Controls	aOR	95% CI		aOR	95% CI		aOR	95% CI		aOR	95% CI		aOR	95% CI	
Cancer	+	88	144	4,6	3,4	6,3	2,8	1,3	6,3	3,7	0,9	15,4	12,6	2,1	77,0			
	-	607	5026	REF			1,1	0,8	1,4	1,3	0,7	2,4	1,5	0,9	2,6	3,3	1,1	10,5
Immobilisation	+	101	82	14,5	10,2	20,6	5,6	1,8	18,1	13,1	2,1	81,9				11,4	0,6	223,1
	-	553	5106	REF			1,1	0,8	1,5	1,7	0,9	3,0	1,9	1,1	3,2	3,5	1,0	12,7
Surgery	+	83	68	11,6	7,9	16,8	19,4	5,5	68,1	6,3	1,0	40,7						
	-	596	5101	REF			1,1	0,8	1,5	1,5	0,8	2,6	2,0	1,2	3,2	4,3	1,4	13,7
COVID-19	+	95	145	6,4	4,7	8,7	3,9	1,1	14,2	12,4	2,0	76,9	7,1	1,4	36,8	10,5	0,5	202,4
	-	577	5031	REF			1,1	0,8	1,5	1,4	0,8	2,5	1,8	1,1	3,0	3,2	0,9	11,7
Any Riskfactor	+	252	380	7,2	5,8	8,8	3,6	1,9	6,8	8,1	3,1	21,2	8,0	2,6	24,5	11,7	0,6	217,9
	-	482	4808	REF			1,3	0,9	1,7	1,3	0,7	2,7	1,8	1,0	3,2	3,7	1,0	13,4

Supplementary Table 2B: Relative risks (Odds Ratios) for the association between venous thromboembolism and several SARS-CoV-2 vaccines within 28 days in several risk groups with risk groups defined by only data from the questionnaire

				No Vaccine			mRNA						Vector					
							BNT162b2 (Pfizer)			mRNA-1273 (Moderna)			AZD1222 (AstraZeneca)			Ad26.COV2.S (Johnson & Johnson)		
		N cases	N Controls	aOR	95% CI		aOR	95% CI		aOR	95% CI		aOR	95% CI		aOR	95% CI	
Cancer	+	116	195	4,4	3,3	5,8	2,2	1,1	4,5	6,5	2,1	19,8	6,1	1,6	23,2			
	-	629	5088	REF			1,1	0,8	1,4	1,3	0,7	2,3	1,6	0,9	2,7	3,3	1,0	10,3
Immobilisation	+	221	99	25,2	18,8	33,8	14,4	6,7	31,1	21,8	4,0	117,7	18,0	4,2	76,6	28,3	2,3	343,5
	-	516	5187	REF			1,2	0,8	1,6	1,9	1,0	3,3	1,7	1,0	3,0	2,4	0,5	11,0
Surgery	+	126	126	9,3	6,9	12,5	7,2	3,0	17,6	8,3	2,0	35,6						
	-	615	5157	REF			1,1	0,8	1,5	1,6	0,9	2,7	2,0	1,2	3,2	4,2	1,3	13,4
COVID-19	+	151	173	8,4	6,5	11,0	11,8	4,3	32,4	12,6	2,0	77,9	7,2	1,4	37,1	10,6	0,5	207,2
	-	595	5112	REF			1,1	0,8	1,5	1,6	0,9	2,8	1,8	1,1	3,1	3,3	0,9	11,9
Any Riskfactor	+	397	519	10,0	8,3	12,1	6,1	3,8	10,0	15,0	6,7	33,7	10,8	4,5	26,2	29,2	2,5	343,9
	-	377	4780	REF			1,3	0,9	1,9	1,0	0,4	2,4	1,9	1,0	3,5	2,9	0,6	13,1

Supplementary Table 2C: Relative risks (Odds Ratios) for the association between venous thromboembolism and several SARS-CoV-2 vaccines within 28 days in several risk groups; each risk factor with missing information imputed as risk factor present

				No Vaccine			mRNA						Vector					
							BNT162b2 (Pfizer)			mRNA-1273 (Moderna)			AZD1222 (AstraZeneca)			Ad26.COV2.S (Johnson & Johnson)		
		N cases	N Controls	aOR	95% CI		aOR	95% CI		aOR	95% CI		aOR	95% CI		aOR	95% CI	
Cancer	+	102	194	3,7	2,8	5,0	2,0	1,0	4,3	5,1	1,6	16,6	4,6	1,1	19,5			
	-	643	5089	REF			1,1	0,8	1,4	1,3	0,8	2,4	1,6	1,0	2,7	3,2	1,0	10,2
Immobilisation	+	208	98	23,3	17,3	31,4	12,8	5,9	28,0	16,8	2,9	96,8	17,7	4,2	75,2	28,2	2,3	341,4
	-	529	5188	REF			1,2	0,8	1,6	1,9	1,1	3,3	1,7	1,0	3,0	2,4	0,5	10,9
Surgery	+	108	125	7,9	5,8	10,7	5,5	2,1	14,1	8,1	1,9	34,3						
	-	633	5158	REF			1,1	0,8	1,5	1,5	0,9	2,6	1,9	1,2	3,1	4,1	1,3	13,0
COVID-19	+	112	167	6,1	4,6	8,0	4,8	1,4	16,0	11,7	1,9	72,2	6,7	1,3	34,5	9,9	0,5	192,5
	-	634	5118	REF			1,1	0,8	1,5	1,5	0,9	2,6	1,7	1,0	2,9	3,0	0,8	11,0
Any Riskfactor	+	349	511	7,9	6,5	9,5	4,5	2,7	7,5	11,1	4,8	25,5	8,5	3,4	21,2	27,4	2,3	324,2
	-	425	4788	REF			1,3	0,9	1,8	1,2	0,6	2,6	1,8	1,0	3,3	2,6	0,6	11,8

Supplementary Table 2D: Relative risks (Odds Ratios) for the association between venous thromboembolism and several SARS-CoV-2 vaccines within 28 days in several risk groups; each risk factor with missing information imputed as risk factor not present

Supplementary Table 3 : Details calculation absolute number of VTE associated with SARS-CoV-2 vaccines

Age	pop NL (18+)	N VTE	% with VTE
<=60	9.568.997	7.562	0,08%
>60	4.597.642	14.854	0,32%
total	14.166.639	22.416	0,16%

	BNT162b2 (Pfizer- BioNTech)							
Age	N vac	%pop	aOR	lci	uci	PAF	lci	uci
<=60	5.613.003	58,7%	1,46	0,9	2,37	4%	-1%	7%
>60	3.071.703	66,8%	0,95	0,58	1,56	-1%	-8%	4%

	mRNA-1273 (Moderna)							
Age	N vac	%pop	aOR	lci	uci	PAF	lci	uci
<=60	889.900	9,3%	0,9	0,28	2,97	0%	-4%	1%
>60	101.487	2,2%	1,98	0,89	4,42	2%	0%	3%

	AZD1222 (AstraZeneca)							
Age	N vac	%pop	aOR	lci	uci	PAF	lci	uci
<=60	299.985	3,1%	1,65	0,47	5,79	1%	-2%	1%
>60	1.000.697	21,8%	1,51	0,75	3,05	1%	-1%	3%

	Ad26.COV2.S (Johnson & Johnson)							
Age	N vac	%pop	aOR	lci	uci	PAF	lci	uci
<=60	728.704	7,6%	3,28	0,8	13,4	1%	0%	1%
>60	11.802	0,3%	-	-	-	0%	0%	0%

Total PAF	Vaccinated					VTE vaccination			% vaccines with VTE		
Age	Total	%	PAF	lci	uci	N	lci	uci	%	lci	uci
<=60	7.531.592	79%	5,1%	-7,4%	10,3%	385	-562	781	0,005%	-0,01%	0,01%
>60	4.185.689	91%	2,6%	-9,6%	9,4%	389	-1429	1402	0,009%	-0,03%	0,03%

	COVID without vaccine								Unvaccinated COVID with VTE					
Age	N	%pop	aOR	lci	uci	PAF	lci	uci	N	lci	uci	% of covid	lci	uci
<=60	1.054.177	11,0%	8,07	5,02	12,97	13,0%	11,9%	13,7%	983	898	1035	0,093%	0,085%	0,098%
>60	244.510	5,3%	11,38	6,58	19,67	11,1%	10,3%	11,5%	1646	1530	1713	0,673%	0,626%	0,700%

	COVID with vaccine								Vaccinated COVID with VTE					
Age	N	%pop	aOR			PAF	lci	uci	N	lci	uci	% of covid	lci	uci
<=60	341.622	3,6%	1,26	0,36	4,39	0,3%	-2,3%	1,0%	20	-171	74	0,006%	-0,050%	0,022%
>60	92.139	2,0%	4,19	1,75	10,04	1,9%	1,1%	2,3%	288	162	340	0,312%	0,176%	0,369%

Vaccination effectiveness for infection				50%				75%				90%
	Extra COVID without vaccination				Extra COVID without vaccination				Extra COVID without vaccination			
Age	N	N VTE	lci	uci	N	N VTE	lci	uci	N	N VTE	lci	uci
<=60	683.244	637	582	671	1.366.488	1274	1164	1342	3.416.220	3184	2910	3354
>60	184.278	1240	1153	1291	368.556	2481	2306	2581	921.390	6202	5766	6454

N VTE because of vaccination in situation with vaccination in NL			
	N	lci	uci
<=60	385	-562	781
>60	389	-1429	1402

N VTE because of COVID in situation without vaccination in NL									
	Vaccine effectiveness 50%			Vaccine effectiveness 75%			Vaccine effectiveness 90%		
Age	N	lci	uci	N	lci	uci	N	lci	uci
<=60	637	582	671	1274	1164	1342	3184	2910	3354
>60	1240	1153	1291	2481	2306	2581	6202	5766	6454

Netto VTE because of vaccines									
Age	N	lci	uci	N	lci	uci	N	lci	uci
<=60	-251	-1233	199	-888	-1903	-384	-2799	-3916	-2130
>60	-851	-2719	248	-2092	-4010	-905	-5813	-7882	-4364

Extra information

Proportion cases exposed

Vaccine		<=60		>60	
28 days		cases	%	cases	%
No vaccine			84%		82%
Pfizer			11%		11%
Moderna			2%		3%
AstraZeneca			2%		4%
Johnson&Johnson			2%		0%
vaccine	covid	<=60		>60	
180-60d	60days	cases	%	cases	%
no	no		41%		45,8%
yes	no		43%		39,5%
no	yes		15%		12,1%
yes	yes		1%		2,5%
**Risks**					
vaccine	covid	<=60		>60	
180-60d	60days	aOR	95CI	aOR	95CI
no	no	1		1	
yes	no	1,53	1.01-2.35	1,19	0.8-1.7
no	yes	8,07	5.0-12.9	11,38	6.6-19.7
yes	yes	1,26	0.4-4.4	4,19	1.7-10.0
